# Understanding the visual function symptoms and associated functional impacts of phakic presbyopia

**DOI:** 10.1186/s41687-021-00383-1

**Published:** 2021-11-03

**Authors:** Sarah Bentley, Amy Findley, Sima Chiva-Razavi, Christel Naujoks, Francesco Patalano, Chloe Johnson, Rob Arbuckle, James S. Wolffsohn

**Affiliations:** 1 Adelphi Values, Cheshire, UK; 2 Novartis Pharma AG, Basel, Switzerland; 3 Health and Life Sciences, Aston University, Birmingham, UK

**Keywords:** Presbyopia, Near vision, Quality of life, Qualitative research, Interviews

## Abstract

**Background:**

Presbyopia is defined as the age-related deterioration in the ability to focus on close objects, causing difficulty with near vision tasks. The study aim was to understand the lived experience of phakic presbyopia and identify all relevant visual function symptoms and associated functional impacts.

**Methods:**

Fifty individuals with clinician-confirmed phakic presbyopia (US n = 30, France n = 10, Germany n = 10) and seven healthcare professionals (HCPs) participated in in-depth, face-to-face, qualitative concept elicitation interviews. Verbatim transcripts were analyzed using thematic analysis methods.

**Results:**

Visual function symptoms reported by participants with phakic presbyopia were categorized as: primary near vision functioning symptoms (impaired near visual acuity, n = 50/50, 100%; difficulty with near vision in dim light, n = 42/50, 84%; difficulty focusing at close distances, n = 30/50, 60%; difficulty seeing things when glare is present, n = 30/50, 60%) and secondary symptoms (eye strain, n = 37/50, 74%; dry eyes, n = 35/50, 70%; headaches, n = 30/50, 60%). Proximal impacts on functional vision included difficulty reading in near vision (n = 49/50, 98%, including printed text and handwriting), seeing objects in near vision n = 48/50, 96%, and performing activities of daily living that require near vision (n = 49/50, 98%, including using a smartphone or computer). Distal impacts on functional vision included emotional, work, financial and social impacts. HCP interviews supported participant findings.

**Conclusion:**

Findings provide a comprehensive understanding of the lived experience of phakic presbyopia which informed the development of a presbyopia conceptual model and patient-reported outcome assessments of vision correction independence and near vision functioning. The sample did not include those whose vision cannot be adequately corrected with lenses or surgery.

**Supplementary Information:**

The online version contains supplementary material available at 10.1186/s41687-021-00383-1.

## Plain English Summary

Presbyopia is worsening of the ability to focus on close objects due to ageing. The objective of this study was to understand the lived experience of presbyopia, specifically to identify the visual symptoms experienced, and how these affect a person’s daily life. Fifty people with presbyopia (from the US n = 30, France n = 10 and Germany n = 10) took part in a face-to-face interview. The interviews took place in three separate rounds. Seven HCPs who specialize in presbyopia also took part in a telephone interview. The key visual symptoms that were reported by people with presbyopia included difficulty seeing close up, difficulty seeing close up in dim light, difficulty focusing at close distances, and difficulty seeing things close up when there is glare. Additional symptoms reported as a result of the key visual symptoms included eye strain, dry eyes and headaches. People with presbyopia reported that these symptoms caused them difficulty to read close up (both printed text and handwriting) and difficulty to see objects in near vision. As a result, people with presbyopia reported a number of other difficulties in their daily living (including using a smartphone or computer), as well as impacts on emotional, work, financial and social aspects of their life. HCPs supported these findings. This study provided an in-depth understanding of the lived experience of presbyopia. The findings supported development of a model of the key symptoms and impacts of presbyopia, and contributed to the development of a questionnaire measuring the ability to do near vision activities.

**Supplementary Information:**

The online version contains supplementary material available at 10.1186/s41687-021-00383-1.

## Introduction

Presbyopia is a condition that causes deterioration in near vision with aging [1, 2]. A systematic review estimated that around 1.8 billion people globally had presbyopia in 2015, with the number likely to increase with the aging population [3]. The condition typically starts to develop in adults around the age of 40 years and is hypothesized to be caused by either a weakening of the ciliary muscles or a loss of lens elasticity preventing focal point change [4, 5]. While the etiology of this condition is not fully elucidated, research suggests that an increase in lens rigidity is the primary causative mechanism [6, 7].

Currently there is no single treatment that reverses the effect of aging on the lens, restoring the ‘true’ dynamic accommodation of the eye [2]. Presbyopia can only be corrected with the use of glasses, contact lenses or refractive surgery, or managed by the use of magnifiers [8], however, there are a number of disadvantages associated with these correction methods [2, 8–10]. It is estimated that around 50% of adults who have presbyopia do not use adequate near correction [3]. The lack of treatment that can restore accommodation in the eye, paired with the limitations of current correction options, means presbyopes continue to experience problems with their sight, highlighting an unmet treatment need [2, 3].

The reduced near visual acuity of presbyopia has a significant impact on individuals’ health related quality of life (HRQoL) [11–14]. Individuals with presbyopia first begin to have difficulty with tasks that require them to see up close, such as reading or threading a needle [15, 16]. Individuals who do not wear glasses or contact lenses may experience headaches and eye strain due to difficulty focusing on objects [17]. As a result of the visual symptoms experienced, individuals with presbyopia have reported an impact to their performance and productivity at work [18]. Despite its commonality and high global prevalence, there has been no formally agreed upon definition of presbyopia [2] and there is a lack of in-depth qualitative research into the lived experience of individuals with presbyopia [15], specifically individuals with phakic presbyopia. Phakic presbyopia refers those individuals who still have a natural lens as opposed to pseudophakic presbyopia where the individual no longer has a natural lens (such as following surgery).

**Fig. 1:**
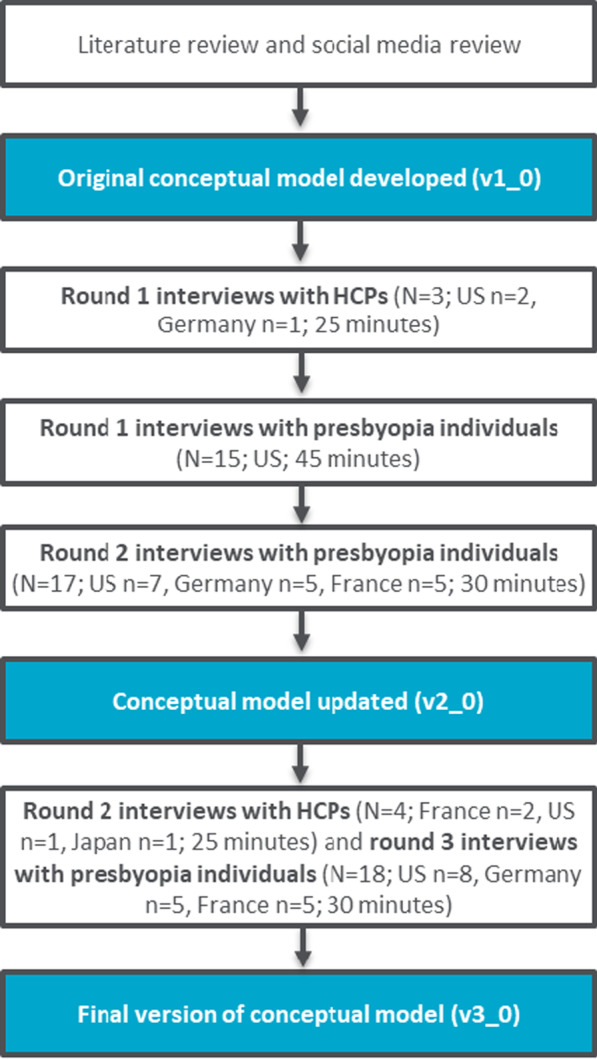
Summary of the study methodology

An eight-step research program was designed to address the gap in the literature (Fig. 1). The first step in the research program included a targeted literature review to identify and evaluate patient-reported outcomes (PRO) assessments used in this population [19]. Key symptoms of presbyopia reported in those articles included: near vision impairment symptoms (difficulties with near vision, blurred vision, difficulties focusing at close distances), and physical symptoms (eye strain, headache, fatigue, dry and irritated eyes). Impacts on HRQoL associated with presbyopia reported in the literature pertain to difficulties reading and seeing things in near vision, an impact on activities of daily living (including using electronic devices), and work, emotional, and social impacts [11–16, 18, 20]. To supplement the literature review and provide further insight into the lived experience of presbyopia, a social media listening study was also conducted during step one of the research program [26]. Findings from social media reports supported those identified in the published literature, including symptoms such as difficulty focusing on near objects and eye strain, and impacts on daily activities, work, and emotional burden due to symptoms.

Although the literature review and social media listening study add to our understanding of presbyopia, much of the peer-reviewed literature that has been published in this area has focused on individuals with diverse refractive errors and therefore the findings are not specific to presbyopia [15]. The aim of the study was to conduct qualitative research to gain a comprehensive understanding of the individual experience of phakic presbyopia and to identify all relevant visual function symptoms and associated functional impacts. A further aim of the study was to develop a conceptual model based on the findings from the literature review, social media listening study, and most importantly this prospective qualitative research.

## Methods

### Study design

This was a non-interventional, qualitative, semi-structured, in-depth, face-to-face, concept elicitation (CE) interview study. As previously noted, this qualitative CE interview study was part of a wider eight-step research study that involved multiple data collection methods (Fig. 1; including a literature review, social media listening study, and qualitative cognitive debriefing interviews to assess the content validity of PRO assessments with individuals who had phakic presbyopia). Only findings from the qualitative CE interviews are reported in this article, findings from CD interviews are reported separately [21].

### Recruitment and eligibility criteria

Ethical approval was obtained prior to the commencement of any study related activities. Fifty individuals aged between 40–64 years with clinician-confirmed phakic presbyopia were recruited in the US (n = 30), France (n = 10), and Germany (n = 10). Recruiting clinicians provided information on the date of diagnosis of presbyopia, visual acuity of each eye, clinician-rated severity of binocular distance-corrected near visual acuity (DCNVA) at 40 cm, and clinician-rated severity of near addition (ADD) to support confirmation of diagnosis. Individuals who had experienced lens extraction or replacement, or those who had an ocular condition which affected their visual acuity (other than short-sightedness [myopia] or long-sightedness [hyperopia]) were excluded from the study. Further information regarding the study inclusion and exclusion criteria can be found in Table 1. Recruitment pre-defined quotas were implemented to ensure insights were gained from a diverse population. This was done by pre-specifying the minimum or maximum number of each demographic or clinical characteristic sub-group that should be targeted for each county.

**Table 1:** Eligibility criteria

Eligibility criteria	
Inclusion criteria	Participants diagnosed with presbyopia aged 40–65 years with confirmation of diagnosis by an optometrist or other healthcare professional
	Fluent speaker, literate and able to read and write in their local language
	Willing and able to provide written informed consent and to perform all study activities; including participating in at least one in-depth interview
Exclusion criteria	Participant has a history of lens extraction or replacement (e.g. cataract surgery)
	Participant has a diagnosis of any other ocular condition, other than short-sightedness (myopia) or long-sightedness (hyperopia), that had an impact on visual acuity (e.g. cataracts)
	Participant has any other physical or mental illness that, in the opinion of the recruiting clinician, might influence the responses they give during the interview or might impact the participant’s ability to engage with the interview or provide appropriate input

Experienced and qualified partner recruitment agencies worked with referring clinicians to recruit individuals who met the inclusion and exclusion criteria. Referring clinicians confirmed the participant’s eligibility by completing a Case Report Form (CRF) and ensured written informed consent was obtained using an Information and Consent Form (ICF) prior to any other study activities and prior to any personal data being shared. Demographic information was collected and individuals with phakic presbyopia were remunerated for taking part.

In addition, seven healthcare professionals (HCPs; ophthalmologists or optometrists) from the US (n = 3), France (n = 2), Germany (n = 1), and Japan (n = 1) were interviewed to obtain a clinical perspective. HCPs were identified by the study sponsor based on their area of expertise and contribution to the field (such as number of publications and congress activity). The research team reached out via email to the identified HCPs to gauge interest in taking part in the study.

### Interview procedure

Individuals with phakic presbyopia were given a unique identification code to anonymize data. This identification code contained information about the individual’s sex, age, presbyopia severity (based on visual acuity reported by the recruiting clinician), and round of interviews they participated in, country, and participant number.

The interviews were conducted face-to-face by trained, experienced interviewers in three rounds between February 2018 and July 2019. A semi-structured interview guide was used which included a mix of open-ended and focused questioning (please see example interview guide questions in Additional file 1 from round one; a shortened version was used during round two and three). Interviewers from Adelphi Values Patient-Centered Outcomes conducted the US interviews in English, while qualified and experienced third party agency interviewers completed the French and German interviews in the local language. Each interviewer received a detailed briefing about the study objectives, protocol and interview guide, highlighting points of critical importance and allowing opportunity for any questions.

Minor updates were made to the interview guide between rounds of interviews to ensure all topics of interest were fully explored. Questioning started with open-ended questions designed to elicit spontaneous comments regarding visual symptoms, impacts on near vision functioning, and distal impacts experienced by individuals as a result of their presbyopia. Individuals with phakic presbyopia were briefly asked about their diagnosis, any adjustments they made to cope with the effects of their symptoms, and their perceptions of current treatment options. Focused probes were used to explore topics of interest that were not mentioned spontaneously in the interview, or to explore concepts that emerged from the open-ended questioning in more detail. All interviews were audio-recorded and lasted between 30–45 min. Participants were renumerated for taking part on completion of the interview.

Interviews with seven HCPs were also conducted to provide clinical input. HCPs did not form part of the research study team. These interviews were used to capture the symptoms and impacts HCPs have observed in individuals with phakic presbyopia. As a key focus of the interview was to obtain insights into the clinical relevance of symptom and impact concepts in phakic presbyopia, more focused probes were used with the HCPs than in the participant interviews. All interviews were conducted by telephone in English and lasted approximately 30 min.

As part of the interview, HCP participants were asked to provide feedback on a draft conceptual model which was developed based on previous research and updated iteratively based on the findings of the successive rounds of interviews conducted as part of this study. Thus, different HCPs provided input on different versions of the conceptual framework. Figure 1 provides an overview of when the conceptual model was updated; HCPs reviewed the latest version of the conceptual model available prior to the interview. HCPs were provided with a monetary renumeration for taking part that was in line with fair market values.

### Data analysis

A qualitative analysis plan (QAP) was developed prior to analysis of the qualitative interview data. All interviews were transcribed verbatim, and the French and German transcripts were translated to English for analysis by an experienced third party agency. Transcripts were imported into Atlas.ti analysis software and qualitatively coded using thematic analysis methods to identify common themes across the data. Adelphi Values Patient-Centered Outcomes team (AF, CJ, SB and RA) analyzed the interview findings. The project lead reviewed the code list and coding applied to the first transcript analyzed by each researcher. Regular meetings were scheduled to ensure consistency in analysis and to reach consensus on any discrepancies.

Thematic analysis is a qualitative analysis method which offers flexibility to provide a rich, detailed and complex synthesis of data that meets a very specific and applied aim [22, 23]. An induction-abduction approach was taken to identifying themes in the data where themes were identified both by topics emerging directly from the data (inductive inference) and by applying prior knowledge from the literature review (abductive inference). A comparative analysis was used to triangulate the inductive and abductive inferences to form a complete list of themes. This enabled the analysis to remain rooted in the data, allowing participants to identify areas of importance for them, but also taking into consideration prior knowledge from previous research. After analyzing each transcript, a list of participant verbatim statements was generated for each coding domain.

Concept frequency was determined by counting the number of participants who mentioned a concept, at least once, during the interview. Data was also captured on how many participants reported a concept spontaneously versus those who discussed it only when probed by the interviewer. Subgroup analyses were conducted to understand whether differences exist in the experiences of individuals with phakic presbyopia according to disease severity (mild vs. moderate/severe), age of participant, country of origin (US vs. France vs. Germany), and presence of myopia. The findings from this stage of analysis were used to support further development of the conceptual model.

Conceptual saturation is recommended by the US Food and Drug Association (FDA) as a method to identify what is important to patients [24], and has been described as the point at which no new insights are likely to be obtained from analysis of further interviews [23, 25]. Conceptual saturation was evaluated by dividing the sample into five equal groups of ten individuals with phakic presbyopia (in the order the interviews were conducted). If no new concepts emerged in the last set of transcripts then it was considered evidence that saturation had been achieved. The findings of this analysis showed that all symptom and impact concepts emerged in the first four sets of transcripts (see Additional file 2). It was therefore concluded that conceptual saturation was achieved and that all concepts had been fully explored within this sample.

Conceptual saturation of the HCP interview findings was not assessed given the small sample size (N = 7), and because HCPs were asked to comment on the conceptual model, potentially biasing responses. Frequency counts were determined for HCP findings; concepts were compared to the findings from interviews with individuals with phakic presbyopia and added to the conceptual model.

## Results

### Sample characteristics

Demographic and clinical characteristics of the participants involved in the study are provided in Table 2. The mean age of the sample was 52.4 years old (range: 40–65) and approximately half of the sample were Caucasian (n = 22/40, 55%; please note that information about race and ethnicity was not collected for the French sample n = 10) and a majority were female (n = 30/50, 60%). Due to the use of quotas, participants were evenly split between those classified as having mild (n = 24/50, 46%) and moderate-severe (n = 26/50, 54%) phakic presbyopia, as reported in their near ADD results provided by the referring physician. Forty-two participants wore glasses for near vision correction (84%), seven wore contact lenses (14%), and one used a magnifying glass when needed (2%).

**Table 2:** Participant demographic and clinical characteristics

Description	France (N = 10)	Germany (N = 10)	USA (N = 30)	Total (N = 50)
Participant demographic characteristics				
**Age (years)**				
Average (range)	55.9 (41–65)	51.1 (40–63)	51.6 (40–65)	52.4 (40–65)
**Sex, n (%)**				
Male	2 (20.0%)	5 (50.0%)	13 (43.3%)	20 (40.0%)
Female	8 (80.0%)	5 (50.0%)	17 (56.7%)	30 (60.0%)
**Race, n (%)**				
Caucasian	*Not appropriate to collect in France*	7 (70.0%)	15 (50.0%)	22 (48.9%)
Black/African American		1 (10.0%)	11 (36.7)	12 (26.7%)
Asian		2 (20.0%)	–	2 (4.4%)
Multi-Racial		–	–	–
Other—Hispanic		–	3 (10.0%)	3 (6.0%)
Missing data		–	1 (3.3%)	1 (2.0%)
**Highest education level, n (%)**				
Some high school	3 (30.0%)	–	2 (6.7%)	5 (10.0%)
High school diploma or GED	–	1 (10.0%)	12 (40.0%)	13 (26.0%)
Some years of college	2 (20.0%)	4 (40.0%)	9 (30.0%)	15 (30.0%)
Certificate program	–	2 (20.0%)	–	2 (4.0%)
University/college degree	3 (30.0%)	2 (20.0%)	6 (20.0%)	11 (22.0%)
Graduate or professional degree	2 (20.0%)	1 (10.0%)	1 (3.3%)	4 (8.0%)
**Work status, n (%)**				
Working full-time	5 (50.0%)	7 (70.0%)	21 (70.0%)	33 (66.0%)
Working part-time	2 (20.0%)	1 (10.0%)	2 (6.7%)	5 (10.0%)
Retired	2 (20.0%)	2 (20.0%)	1 (3.3%)	5 (10.0%)
Full-time homemaker	1 (10.0%)	–	3 (10.0%)	4 (8.0%)
Looking for work	–	–	1 (4.6%)	1 (2.0%)
Not working due to another illness	–	–	1 (3.3%)	1 (2.0%)
Missing data	–	–	1 (3.3%)	1 (2.0%)
**Participant self-reported severity of phakic presbyopia, n (%)**				
Very severe	1 (10.0%)	1 (10.0%)	1 (3.3%)	3 (6.0%)
Severe	1 (10.0%)	3 (30.0%)	6 (20.0%)	10 (20.0%)
Moderate	7 (70.0%)	–	14 (46.7%)	21 (42.0%)
Mild	1 (10.0%)	6 (60.0%)	9 (30.0%)	16 (32.0%)
**Participant clinical characteristics (reported by recruiting clinician)**				
Years since diagnosed, average (range)^a^	10.4 (0.5–20.9)	7.3 (1–17.1)	7.6 (0.2–34.6)	8.1 (0.2–34.6)
Visual Acuity score of left eye (decimal), average (range)^1^	0.92 (0.6–1.0)	0.67 (0.5–0.8)	0.63 (0.2–1.0)	0.69 (0.2–1.0)
Visual Acuity score of right eye (decimal), average (range)^1^	0.92 (0.6–1.0)	0.64 (0.4–0.8)	0.62 (0.3–1.0)	0.69 (0.3–1.0)
**Severity of participants’ binocular DCNVA at 40 cm, n (%)**				
Mild	2 (20.0%)	6 (60.0%)	14 (46.7%)	22 (44.0%)
Moderate-severe	3 (30.0%)	4 (40.0%)	16 (53.3%)	23 (46.0%)
Information not available	5 (50.0%)	–	–	5 (10.0%)
**Severity of participants’ near ADD, n (%)**				
Mild	3 (30.0%)	6 (60.0%)	14 (46.7%)	23 (46.0%)
Moderate-severe	7 (70.0%)	4 (40.0%)	16 (53.3%)	27 (54.0%)
**Clinician reported myopia/near sightedness**†**, n (%)**				
None	6 (60.0%)	5 (50.0%)	18 (86.4%)	29 (58.0%)
Mild	2 (20.0%)	–	2 (6.7%)	4 (8.0%)
Moderate	1 (10.0%)	–	2 (6.7%)	4 (8.0%)
High	1 (10.0%)	–	3 (10.0%)	4 (8.0%)
Missing data	–	5 (50.0%)	5 (4.5%)	10 (20.0%)
Concomitant conditions, n (%)*				
** Yes:**	1 (10.0%)	–	2 (6.7%)	3 (6.0%)
Posterior detachment of the left vitreous	1 (10.0%)			1 (2.0%)
Asthma			1 (3.3%)	1 (2.0%)
Glaucoma			1 (3.3%)	1 (2.0%)
COPD			1 (3.3%)	1 (2.0%)
**Current treatment for phakic presbyopia, n (%)**				
** Glasses ~ :**	7 (70.0%)	5 (50.0%)	22 (73.3%)	34 (68.0%)
Unspecified	1/7 (14.3%)	5/5(100%)	14/22 (63.6%)	20/34 (58.8%)
Single vision	2/7 (28.6%)	–	4/22 (18.2%)	6/34 (17.6%)
Multifocal	4/7 (57.1%)	–	2/22 (9.1%)	6/34 (17.6%)
Bifocals	–	–	2/22 (9.1%)	2/34 (5.9%)
**Contact lenses**	–	5 (50.0%)	2 (6.7%)	7 (14.0%)
**Myopia treatment reported only**	1 (10.0%)	–	3 (10.0%)	4 (8.0%)
**Missing data^**	3 (30.0%)	–	5 (16.7%)	8 (16.0%)

^*^Multiple answers possible. †Clinicians were not asked to confirm diagnosis of myopia for the round 1 interviews. ^1^Seven participants data missing. ~ Clinicians reported multiple types of glasses for some participants (specifically two types of glasses for two participants, and three types of glasses for one participant). Additionally, all but four participants reported using glasses during the interviews.

^a^ One participant’s data was removed in this category only as it appeared to have an error.

^Of note, one participant reported that they only used magnifiers during the interview and no other form of vision correction aid

With regards to the HCP sample, five HCPs were ophthalmologists and two were optometrists. All HCPs had more than 10 years of experience managing individuals with presbyopia and five reported treating more than 31 individuals with presbyopia each month. HCPs reported seeing each individual with presbyopia for a routine appointment once per year (n = 3), twice per year (n = 2), or monthly (n = 2).

#### Participant findings

##### Symptoms reported by individuals with phakic presbyopia

**Fig. 2:**
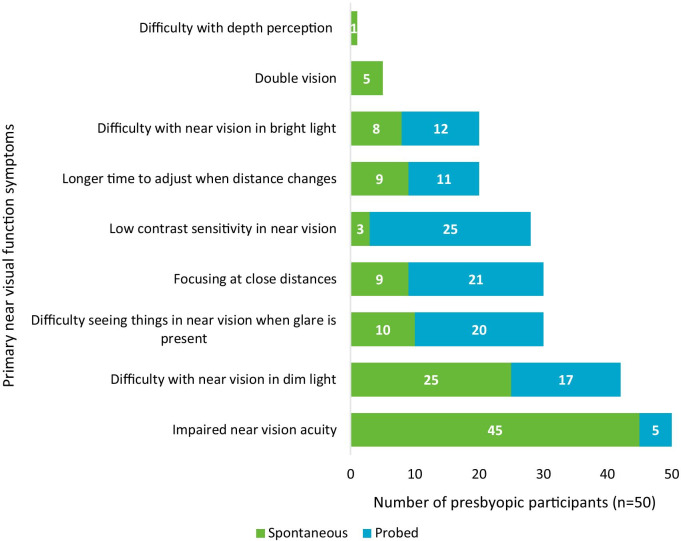
Overview of primary near vision function symptoms reported by individuals with phakic presbyopia

*Visual function symptoms* Visual function symptoms reported by participants with phakic presbyopia (see Fig. 2) were categorized as either primary near vision functioning symptoms or secondary symptoms. All participants reported impaired vision acuity as a result of presbyopia (n = 50/50, 100%). When describing their near vision acuity, participants commonly used a variation of the term ‘blurry’ (n = 23/50, 46%) or referred to ‘difficulty seeing’ (n = 17/50, 34%); for instance one participant described their near vision acuity as ‘glassy’:

Uh, a lot of some other symptoms are, uh, I guess I would call it like a glassy vision. It’s almost as if my vision is if, uh, I’d been swimming for hours. Like sort of everything appears to have like a reflective, uh, rainbow hue to it. (M44-MOD-US7).

Most participants reported that they experienced impaired near vision in dim light (n = 42/50, 84%), such as difficulty reading in low light (n = 13/42, 31%) and seeing close-up in dark environments (n = 12/42, 29%). For example one participant reported challenges with dining out in a dimly lit restaurant:

Um, it makes, you know, dining out in dimly lit restaurants is difficult, uh, whereas I may be able to read that print, um, if it was brightly lit. I can’t when it’s dimly lit. (F52-MOD-US1).

A large proportion of participants described difficulty focusing at close distances (n = 30/50, 60%), difficulty seeing when glare is present (n = 30/50, 60%), low contrast sensitivity (n = 28/50, 56%), difficulty with near vision in bright light (n = 20/50, 40%), and taking longer for their eyes to adjust focus when the distance of vision changes (n = 20/50, 40%). More detail on each visual function symptom is provided in Additional file 3.

*Physical (secondary) symptoms* Several secondary symptoms were described by participants; most commonly eye strain (n = 37/50, 74%), dry eyes (n = 35/50, 70%), headaches (n = 30/50, 60%), eye irritation (n = 18/50, 36%), tired eyes (n = 17/50, 34%), and fatigue (n = 12/50, 24%).

Of those who were asked (n = 43/50, 86%), the most bothersome symptom was difficulty with near vision (n = 12/43, 28%), followed by headaches (n = 7/43, 16%) and seeing in dim light (n = 5/43, 12%). Around a third of participants reported that the reliance on vision correction methods (e.g., glasses and contact lenses) was the most bothersome characteristic of phakic presbyopia (n = 16/43, 37%), with one participant stating:

Um, just the fact that I'm relying on glasses. I mean just that alone just kind of scares me because it's been getting—it's getting progressively worse. You know, I just can't seem to function without glasses (M50-MOD-R2-US8).

##### Impacts reported by individuals with phakic presbyopia

Impacts on functional vision reported by individuals with phakic presbyopia were broadly categorized as proximal impacts (those directly related to near vision impairments) and distal impacts (those occurring as a result of the proximal impacts). Proximal impacts on functional vision of phakic presbyopia largely focused on activities of daily living that were impacted by near vision impairments associated with phakic presbyopia. Distal impacts included emotional impacts, work impacts, social impacts, and financial impacts resulting from the proximal impacts. Participants also described the burden of using current corrective methods for phakic presbyopia, namely glasses and contact lenses.

##### Proximal functional impacts

**Fig. 3:**
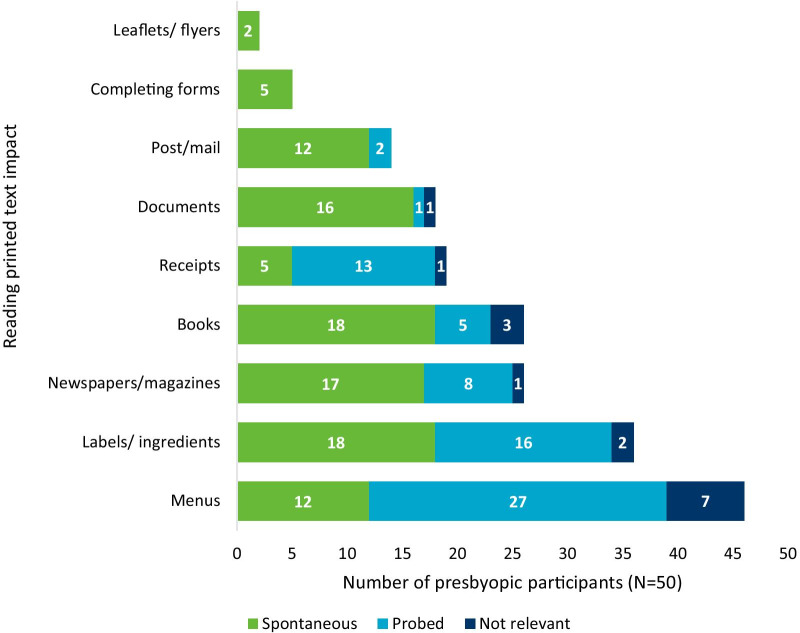
Reading printed text impacts reported by individuals with phakic presbyopia

*Reading small print* All but one participant reported difficulty with near vision reading (n = 49/50, 98%). This included difficulty reading printed (n = 49/49, 100%) and handwritten (n = 34/49, 69%) text. The most commonly reported impacts of difficulty reading printed text (see Fig. 3) included difficulty reading menus (n = 39/49, 80%) and difficulty reading labels or ingredients (n = 34/49, 69%).

*Seeing objects in near vision* Most participants reported difficulty seeing objects close to them (n = 48/50, 96%), such as difficulty reading a wristwatch (n = 14/48, 29%), doing cosmetic tasks (e.g., applying make-up, tweezing eyebrows, and polishing nails; n = 5/48, 10%), and seeing objects in a store (n = 4/48, 8%). One participant, for example, reported difficulty seeing things that are right in front of him, despite wearing his glasses:

I wear the glasses. But despite these glasses, for example when I go shopping, I often see things that are right in front of me, not at all (M63-MOD-R1-DE1).

*Daily living impacts* All but one participant (n = 49/50, 98%) described the impact phakic presbyopia has on their ability to do daily activities that rely on near vision. Participants reported challenges when using digital devices (n = 48/49, 98%), such as a cellphone (n = 46/49, 94%) or a computer (n = 43/49, 88%). Five participants (n = 5/49, 10%) reported that they struggled to use their computer for extended periods of time, indicating that doing so would give them headaches or blurred vision:

Um, I’ve been using computers, I’ve been working with computers for like 10 years so now they’re getting worse, um, because I’m up on the screen so now I really, really have to continuously use my glasses but sometimes I get the headaches, um, the blurry vision (F42-MOD-R2-US3).

Participants reported that phakic presbyopia affected their ability to drive (e.g. seeing the dashboard; n = 30/49, 61%), perform precision work such as sewing (n = 25/49, 51%), cook (n = 20/49, 41%), use a wristwatch (n = 20/49, 41%), shop (n = 16/49, 33%), participate in sports and exercise (n = 14/49, 29%; e.g. swimming, skiing, cycling), engage in hobbies (n = 14/49, 29%; e.g. arts and crafts, coin or stamp collecting, completing puzzles/board games, going to the cinema), or handwrite with a pen or pencil (n = 12/49, 25%). More detail regarding proximal impacts of phakic presbyopia reported by participants is provided in Additional file 3.

*Adjustments to phakic presbyopia* To cope with living with phakic presbyopia, participants reported the use of glasses (n = 18/50, 36%), closing their eyes or taking a break (n = 4/50, 8%), or carrying out certain activities in bright lighting conditions (n = 3/50, 6%). One participant, for instance, reported the importance of better lighting to help with their near vision acuity:

So you really have to get under better lighting and then you zoom in on it. (M54-MILD-US12).

To compensate for difficulty reading, participants reported several adjustments including adjusting the distance from the text (n = 10/49, 20%), asking someone to read the text to them (n = 9/49, 18%), and using a magnifying glass to enlarge the text (n = 7/49, 14%). In addition, participants adopted several strategies to help them see text and objects on their digital devices such as increasing the font size (n = 32/49, 65%), zooming in on the text or object (n = 21/49, 43%), and increasing the brightness on their device (n = 13/49, 27%).

##### Distal impacts

*Emotional impacts* As a result of phakic presbyopia, participants reported several distal impacts on functional vision; the most frequently reported impact was how phakic presbyopia made them feel emotionally (n = 39/50, 78%). Participants described how they often felt frustrated, irritated, aggravated, or disappointed (n = 21/39, 54%) when they were unable to read something:

…it kind of frustrates me because I can’t read some things I need to read (M55-MILD-US5).

Participants reported feeling old (n = 20/39, 51%; as change or loss of vision is often associated with the elderly), scared, worried, or insecure (n = 11/39, 28%). One participant, for example, mentioned that she found it particularly frightening when she is unable to read the label on her medication:

Medication directions. I think that’s really – that’s scary when you can’t read that (F54-MILD-R1-US5).

Other emotional impacts included feelings of sadness, depression, and distress (n = 11/39, 28%; about not being able to read content), inconvenience (n = 10/39, 26%), annoyance (n = 10/39, 25%; towards the condition) and a loss of independence (n = 6/39, 15%).

*Impact on work* Participants reported that phakic presbyopia has an impact on their ability to do their work (n = 36/50, 72%) including difficulty seeing content on their work computer (n = 18/36, 50%), reading documents (n = 17/36, 47%), and performing precision work (n = 10/36, 28%). As a result, several participants reported a loss in productivity (n = 11/36, 31%) due to taking more time to complete work tasks:

Um, sometimes just time loss, you know, if you think time/money quotient in terms of efficiency, in terms of productivity… (M45-MOD-R1-US).

Further, one participant mentioned that she had to take time off work because of phakic presbyopia, while another mentioned that phakic presbyopia could have contributed to his job loss.

*Social impacts* Several participants reported that having phakic presbyopia has affected them socially (n = 19/50, 38%), such as the need to ask others for help (n = 5/19, 26%; e.g., reading labels), difficulty recognizing faces (n = 5/19, 26%), and participating in social activities that require reading (n = 4/19, 21%; e.g., eating at a restaurant or playing games). Two participants specifically mentioned the impact phakic presbyopia has on their ability to read or play with their child or grandchildren, and a further two participants described how it affects them when other people notice them having difficulty reading text.

*Financial impact* One-third of participants reported being financially impacted by phakic presbyopia (n = 17/50, 34%). The greatest expense was the need to purchase glasses (n = 13/17, 77%). Further, a few participants also mentioned the impact of phakic presbyopia on income security, including needing to take sick leave (n = 1/17, 6%), loss of productivity (n = 1/17, 6%), and potential loss of employment due to the condition (n = 1/17, 6%).

*Impact of current correction options* Participants described the burden of near vision correction aids, such as glasses and contact lenses, such as reliance on glasses (n = 30/50, 60%), the need to frequently take glasses on and off (n = 21/50, 42%), the need to carry glasses with them at all times (n = 15/50, 30%), and the need for multiple pairs of glasses (n = 11/50, 22%). Other burdens reported by four to eight participants included: having to clean glasses, not wanting to wear glasses for cosmetic reasons, frequent adjustments to prescription, dislike of bifocals, dislike of wearing glasses, compromised vision, difficult adjusting to glasses, and discomfort of wearing glasses. Eye irritation or dryness (n = 4/50, 8%) and fear of touching the eye (n = 4/50, 8%) were the most commonly reported burdens of using contact lenses. Other burdens reported by three participants or less included: difficulty taking contacts in and out, compromised vision, unable to leave in for a long period of time, the contacts becoming dirty, a specific medical incident involving contact lenses, and contacts being too complicated to use.

##### Subgroup differences in symptoms and impacts

Differences in primary near vision functioning symptoms across sub-groups were minimal. Difficulty focusing at close distances was more common among older participants, particularly among those over the age of 60 (n = 9/11, 82%), likely due to greater severity of phakic presbyopia. Participants with moderate-severe phakic presbyopia (n = 19/27, 70%) were more likely to have difficulty seeing when glare is present compared to those with mild phakic presbyopia (n = 11/23, 48%), which is in line with findings from the HCP interviews that this symptom may be associated with more severe forms of phakic presbyopia (described in section titled ‘HCP findings’). Similarly, a larger proportion of participants with moderate-severe phakic presbyopia (n = 14/27, 52%) reported difficulty with near vision in bright light compared to those with mild phakic presbyopia (n = 6/23, 26%), suggesting that it may be a symptom that becomes worse with greater severity. Minimal differences between individuals who experienced myopia and those who did not were observed, the main difference being that only those who did not have myopia reported a treatment impact of having difficulty adjusting to using glasses. No noticeable differences were found between males and females, or between participant subgroups for proximal or distal impacts of phakic presbyopia.

#### HCP findings

For the most part, HCPs discussed concepts similar to those reported by individuals with phakic presbyopia. However, in discussing these concepts, HCPs often provided clinical reasons for the symptoms and associated impacts that individuals with presbyopia typically experience. HCP reported symptoms and impacts of presbyopia are described below. Of note, four of the HCPs reported that they themselves had presbyopia and two HCPs reported that they did not have presbyopia. One HCP did not report whether they experienced presbyopia themselves or not.

##### HCP reported symptoms of presbyopia

**Fig. 4:**
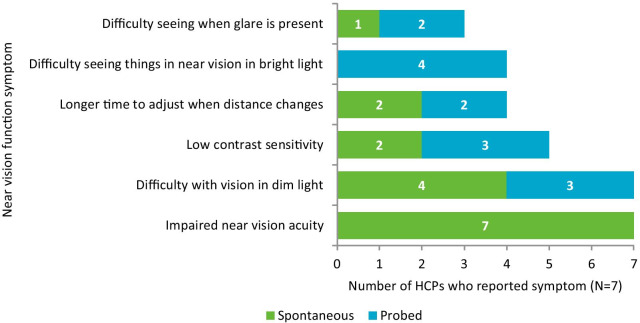
Overview of near vision symptoms reported by HCPs

The HCPs discussed six key concepts relevant to near vision function symptoms (Fig. 4): impaired near vision acuity (n = 7/7, 100%), difficulty with vision in dim light (n = 7/7, 100%), low contrast sensitivity (n = 5/7, 71%), longer time to adjust when distance changes (n = 4/7, 57%), difficulty with vision in bright light (n = 4/7, 57%), and difficulty seeing in near vision when glare is present (n = 3/7, 43%).

The HCPs interviewed also discussed five secondary symptoms. The most frequently discussed secondary symptoms were headaches (n = 5/7, 71%) and fatigue (n = 5/7, 71%). According to one HCP, headaches were often the result of intense focus, stating:

They need to focus more so they will accommodate more and excessive accommodation can lead to some headaches (HCP7).

Other secondary symptoms mentioned by HCPs were eye strain (n = 3/7, 43%), eye irritation (n = 3/7, 43%), and dry eyes (n = 2/7, 29%); however, a few HCPs mentioned that eye irritation and dry eyes are often not necessarily a direct symptom of presbyopia, but can be co-morbid with other eye conditions.

##### HCP reported impacts of presbyopia

All seven HCPs interviewed described the impact that presbyopia has on an individuals’ ability read text, see objects, and use digital devices. In particular, they described the difficulty that individuals with presbyopia have with reading text, in both paper and digital form, due to their inability to see text in near vision or in small print. As one HCP reported:

Whether that means reading a computer, laptop, office-based computer, cell phone, magazine, book. Reading is probably the most universal near task that people are troubled by losing or having difficulty with (HCP2).

As a consequence of this loss in near vision, HCPs all described instances where individuals with presbyopia held objects farther away in order to see them (n = 7/7, 100%), with one HCP noting that sometimes individuals would say “*that their arms are too short to see things…*” (HCP2). Five HCPs described adaptations that individuals with presbyopia may make to help them read or see better on digital devices (n = 5/7, 71%), including increasing the brightness of the screen, zooming in, and enlarging font (n = 2/5, 40%); using the flashlight on their phone to create more light (n = 2/5, 40%), and turning the phone horizontally, moving the device further away from them, getting a larger model of a phone, or using a plug-in keyboard (n = 1/5, 20%).

In line with this, six HCPs reported that presbyopia impacts individuals’ work (n = 6/7, 86%), including the ability to use a computer (n = 6/6, 100%) and read documents (n = 2/6, 33%), as well as deliver presentations or move between working on and off a computer (n = 2/6, 33%). For instance, one HCP reported on the difficulty individuals with presbyopia can experience when switching between working on a computer and looking at a piece of paper or a colleague across a table:

like they’ll talk about having to go back and forth and look at the computer all day and then look at a piece of paper next to it at different distances and then, you know, look at people across a table or in a conference or meeting and presenting things (HCP1).

HCPs also reported that presbyopia can affect individuals’ ability to engage in a number of other activities of daily living, including hobbies, such as sewing or needlework (n = 3/7, 43%), sports (n = 2/7, 29%), writing (n = 2/7, 29%), driving (n = 2/7, 29%), reading a wrist watch (n = 2/7, 29%), applying make-up (n = 1/7, 14%), counting money (n = 1/7, 14%), and travel (n = 1/7, 14%). For instance, HCP2 reported on how driving can be affected by presbyopia by making it difficult to read a map or navigation system:

So your seeing signs isn’t a problem, but reading a map might be. Or looking at your console to know where you are if you have a navigation system in your car (HCP2).

Here, the HCP makes a distinction between reading street signs, which typically require far sightedness, and maps or navigation systems, which require the ability to see in near vision.

Presbyopia can also take an emotional toll on individuals with the condition; five HCPs (n = 5/7, 71%) reported that individuals with presbyopia often express feelings of frustration, feeling old, grief, feelings of sickness, annoyance, and loss of self-esteem, confidence, and independence. For example, one HCP mentioned the frustration individuals often feel when they are no longer able to engage in specific activities:

So, there’s definitely a lot of frustration with not being able to do both their work activities and then hobbies and things they enjoy (HCP1).

Two HCPs (n = 2/7, 29%) mentioned that presbyopia can have a social impact, affecting individuals’ ability to recognize faces, participate in social activities that involve reading content in near vision, and engage in conversations.

The impact of wearing glasses for vision correction was mentioned by five HCPs (n = 5/7, 71%), with three (n = 3/7, 43%) explaining that some individuals with presbyopia do not like to wear them because of how they look:

It’s that they probably just don’t want a pair of glasses on their face, either for cosmetic reasons or convenience (HCP1).

Dependency on the use of glasses to function was reported by three HCPs (n = 3/7, 43%) as an annoyance expressed by individuals with presbyopia:

…that’s what they see as a big problem, is the necessity to always be dependent on some specs to read (HCP7).

Related to dependency was the need to continually swap between different types of glasses (i.e., reading and distance) (n = 2/5, 40%), the need to take glasses on and off (n = 1/5, 20%), and the need for multiple pairs of glasses (n = 1/5, 20%). Further, one HCP (n = 1/5, 20%) reported that glasses can compromise vision as near vision glasses can restrict middle or far distance vision, and one HCP mentioned how individuals can find it uncomfortable to wear glasses if they are not used to wearing them. Additionally, three HCPs described impacts of wearing contact lenses for vision correction (n = 3/7, 42.9%), including compromising distance vision (n = 2/3, 67%), problems with depth perception (n = 1/3, 33%), and risk of infection (n = 1/3, 33%).

#### Development of a conceptual model for phakic presbyopia

**Fig. 5:**
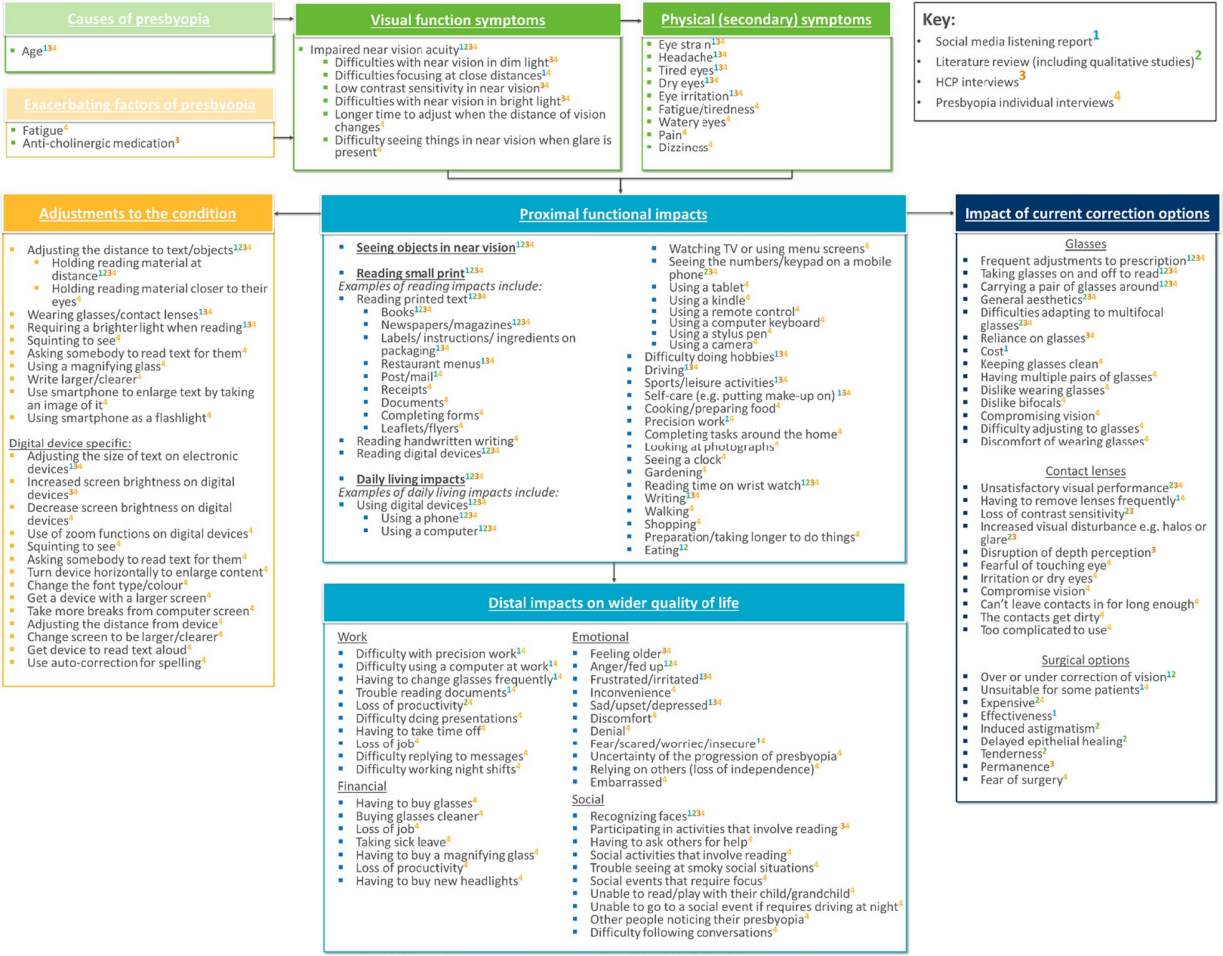
Conceptual model

The preliminary conceptual model developed based on findings from the literature review and social media listening study was iteratively updated at each stage of the research (see Fig. 5 for the final version of the conceptual model). The key provided in the conceptual model indicates which concepts were identified from which source. HCPs provided feedback on the conceptual model during their interviews. All HCPs felt that the version of the conceptual model they reviewed was an appropriate representation of phakic presbyopia and only minor modifications were made based on their feedback. A number of additional concepts were added to the preliminary conceptual model following each round of interviews with participants with phakic presbyopia.

HCP feedback on the conceptual model led to the exclusion of ‘excessive use of digital displays’ as a cause of phakic presbyopia. Some HCPs commented that difficulties in bright light or when glare is present were not necessarily phakic presbyopia-specific, and could be related to other visual conditions (e.g. cataracts); however, these concepts were reported by participants with phakic presbyopia and were therefore retained. Additionally, some HCPs mentioned that dry eyes and irritation could be due to age or other visual conditions. However, these concepts were retained since these were reported in the literature review and by participants with phakic presbyopia in the qualitative interviews.

The HCPs responded positively to the proximal and distal impacts sections of the conceptual model. Two HCPs in round one recommended removing ‘eating’ from the impacts section and this was also not discussed by participants with phakic presbyopia, however this was retained as it was identified in both the social media listening study and the literature review. One HCP recommended removing ‘watching TV’, ‘walking’, ‘driving’, and ‘gardening’ from the conceptual model as they did not regard them as near vision activities, however these were all mentioned by participants with phakic presbyopia, but could be regarded as potentially less relevant than other concepts.

## Discussion

Presbyopia has a long research history. Despite this, there is a lack of qualitative evidence of individuals’ subjective experience of the condition. Most of the literature that exists on HRQoL in related ocular conditions has focused on individuals with diverse refractive errors, providing insights that are not specific to the experience of phakic presbyopia [15]. To fill this gap, the present study examined the lived experience of phakic presbyopia through CE interviews with individuals with a confirmed diagnosis and HCPs (such as ophthalmologists and optometrists) who managed individuals with phakic presbyopia and could provide clinical insights.

Findings from the study highlight that phakic presbyopia is characterized by primary near vision function symptoms (e.g., reduced near vision acuity and specific aspects of this impairment) and secondary symptoms (e.g., dry eyes), which have a significant impact on an individual’s ability to read in near vision, see objects close up, and engage in activities of daily living that require near vision. Related to this, individuals with the condition often experience emotional (e.g., stress, sadness, frustration), social (e.g., difficulty recognizing faces), and financial (e.g., purchasing glasses) impacts, occurring as a result of the symptoms and proximal impacts. Current vision correction options, namely glasses and contact lenses, can be viewed as costly and burdensome.

Findings reported in this study are consistent with previous published literature in this area, highlighting the significant impact on individuals’ HRQoL due to reduced near visual acuity [11–14]. However, the present study adds more depth on most concepts of interest. Key symptoms of near vision impairment and physical symptoms reported in the literature were also described by individuals with phakic presbyopia and HCPs in this study, such as impaired near vision acuity and eye strain. Furthermore, the impacts on HRQoL associated with phakic presbyopia identified in the present study, such as difficulties reading and seeing things in near vision, activities of daily living (such as difficulty using digital devices and driving), and work, emotional, and social impacts, have also been reported to some degree in the literature [11–16, 18, 20]. While key near vision function symptoms and proximal impacts reported in the qualitative interviews were largely in line with concepts reported in the literature, this study provides a more comprehensive understanding of the individual experience of phakic presbyopia by identifying all relevant near vision functioning symptoms and associated functional impacts that are important to individuals and providing in-depth accounts of each concept. In particular, the qualitative interviews yielded more concepts than have been reported in the literature to date, such as certain visual function symptoms (including difficulty seeing up close after looking farther away and difficulty seeing up close when glare is present), and proximal impacts (including difficulty reading different types of materials such as receipts and use of digital devices such as a remote control and computer keyboard). Furthermore, financial impacts were described and additional social, emotional, and work concepts were identified, thus providing a more holistic representation of the experience of individuals with phakic presbyopia.

Confidence that all concepts have been fully explored and that an in-depth understanding of the lived experience of phakic presbyopia has been achieved was supported by the large qualitative sample of individuals with a confirmed phakic presbyopia diagnosis. Saturation analysis provides evidence that saturation was achieved, further supporting that an adequate sample size was used and that no further interviews were required to understand the individual experience of phakic presbyopia. In addition, the inclusion of individuals with phakic presbyopia from three countries who range in age, sex, race, and disease severity provides some confidence that the findings are representative and generalizable within similar cultures and specific countries. The transferability of the findings to non-Western contexts, however, may be limited as all individuals with phakic presbyopia who participated in this study were from Western countries in highly developed nations. It is possible experiences of phakic presbyopia may differ in non-Western contexts due to variation in culture and day-to-day tasks. Future research should therefore be conducted in Asian countries as well as in South America and Africa to provide further evidence regarding the degree to which the findings reported in this study can be generalized cross-culturally and within specific countries. Of note, this study sample did not include those whose vision cannot be adequately corrected with lenses or surgery. Although not specifically probed during the interviews, when comparisons were drawn between the experience of phakic presbyopia between males and females, there were no apparent differences.

The findings from this study have been captured in a conceptual model for phakic presbyopia, which is valuable for facilitating in-depth understanding of the phakic presbyopia-specific individual experience. The model has applications for both clinical practice and clinical research. In the context of this study, the conceptual model has been used to inform the development and refinement of Patient Reported Outcome (PRO) measures assessing vision correction independence and near vision functioning for use in phakic presbyopia clinical trials, in line with FDA guidance [27]. The conceptual model could further be used to determine which PROs to use in clinical trials for phakic presbyopia to cover significant individual experiences relevant to treatment outcomes.

The findings also have implications for clinical practice. Practicing ophthalmologists and optometrists can refer to the findings and resulting conceptual model to increase their understanding of individual experience of phakic presbyopia and the ways that phakic presbyopia can affect an individual’s HRQoL.

## Conclusion

In conclusion, evidence from this qualitative interview study provides a comprehensive understanding of the lived experience of phakic presbyopia and the impact that phakic presbyopia has on near vision functioning and HRQoL. Concepts pertaining to vision function symptoms and associated impacts of phakic presbyopia have been summarized in a conceptual model, which can be used to guide the development of future therapies as well as by practicing ophthalmologists and optometrists to gain an understanding of the phakic presbyopia-specific experience and how phakic presbyopia affects individuals’ HRQoL. The findings have informed the development of PRO assessments of vision correction independence and near vision functioning for use in phakic presbyopia clinical trials. Future research should examine the degree to which the findings are generalizable to non-Western contexts.

## Supplementary Information

**Additional file 1**. Interview guide for individuals with presbyopia.

**Additional file 2**. Conceptual saturation.

**Additional file 3**. Additional primary near vision functioning symptom findings.

## Data Availability

The data that support the findings of this study are available from Novartis Pharma AG but restrictions apply to the availability of these data, which were used under license for the current study, and so are not publicly available. Data are however available from the authors upon reasonable request and with permission of Novartis Pharma AG.
