# Supplementary figures and images for: Understanding the visual function symptoms and associated functional impacts of phakic presbyopia
Source: J Patient Rep Outcomes. 2021 Nov 3;5:114. doi: 10.1186/s41687-021-00383-1 (PMC8566618; doi:10.1186/s41687-021-00383-1)

# Appendices

## Appendix A


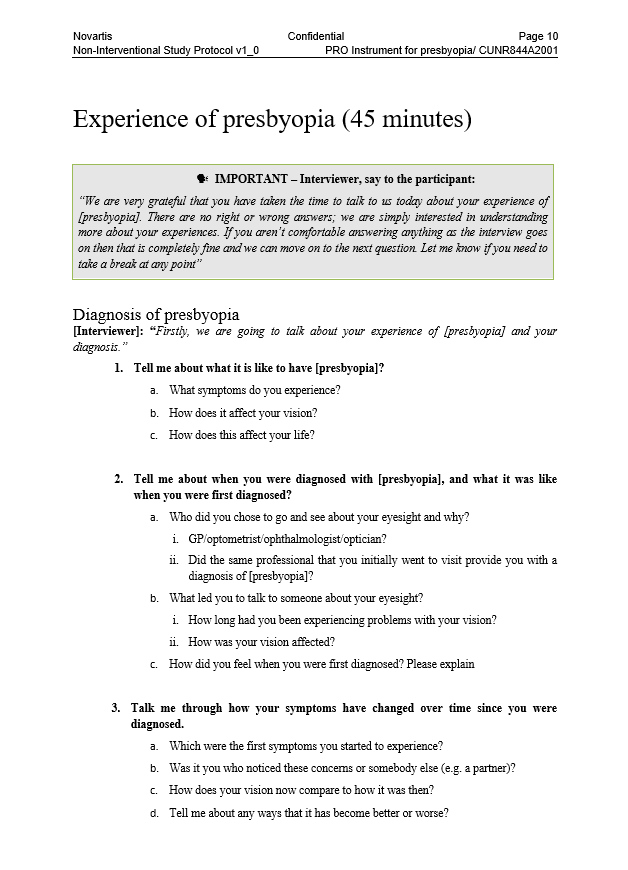


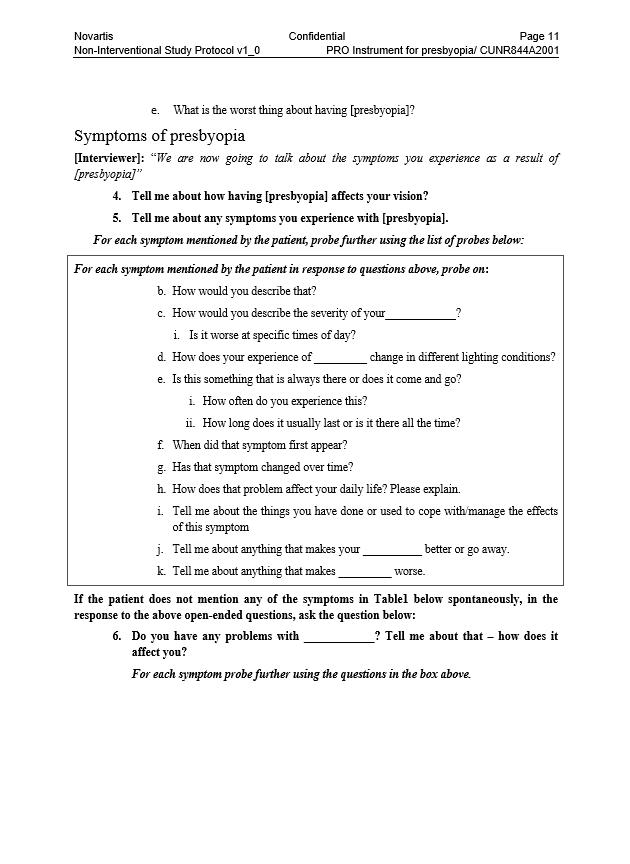


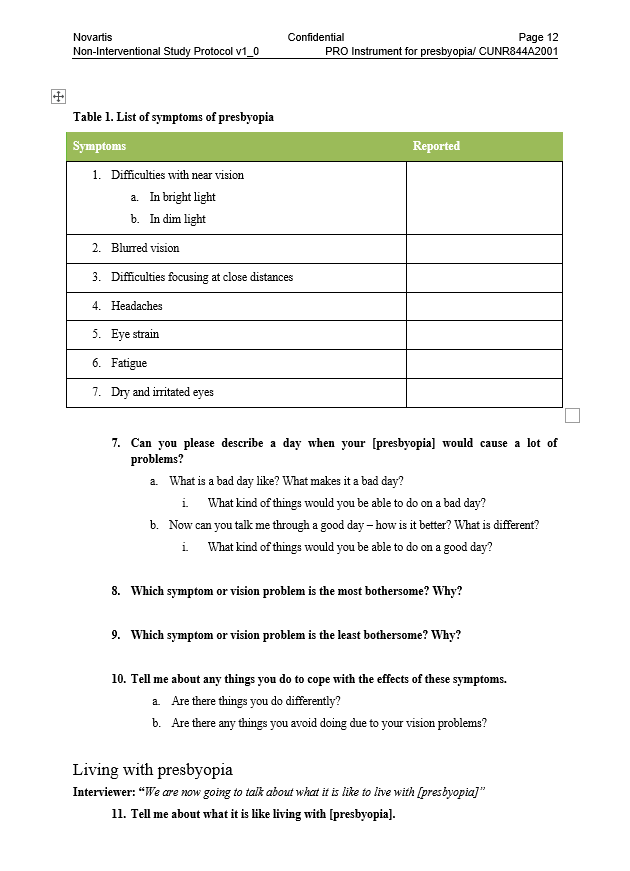


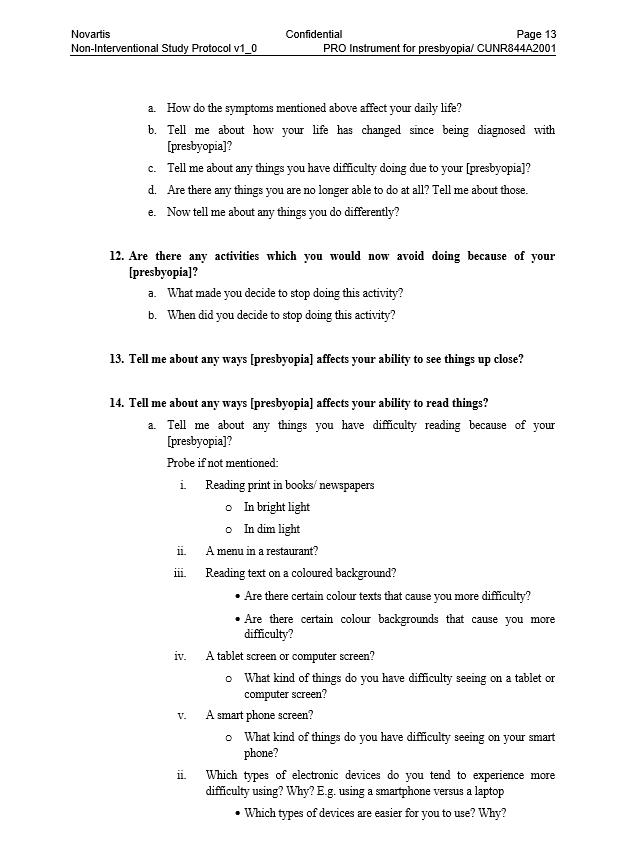


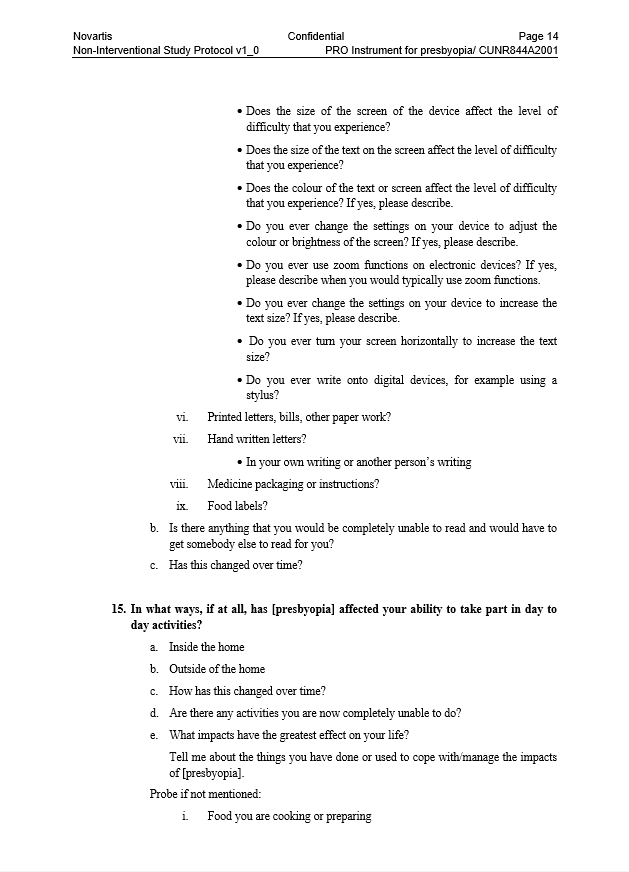


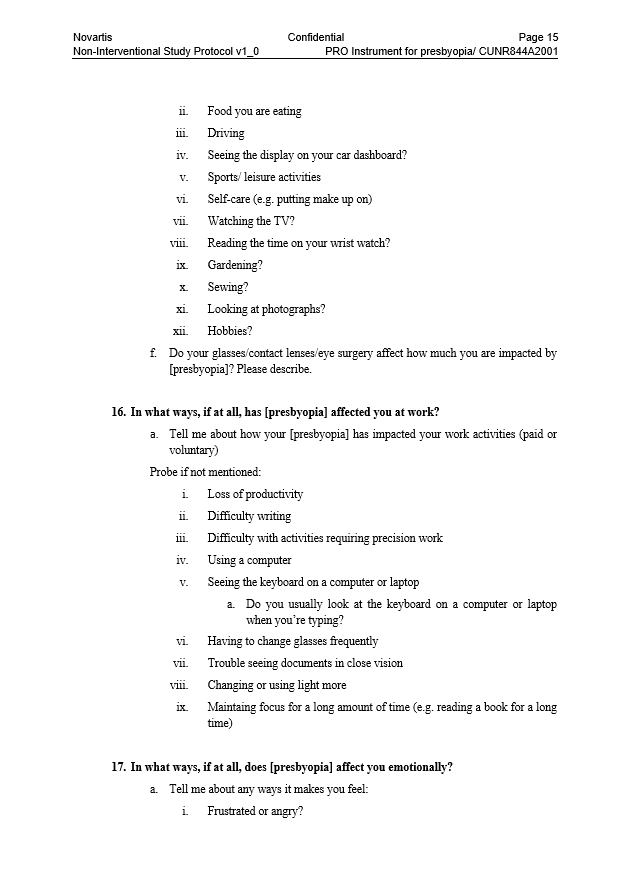


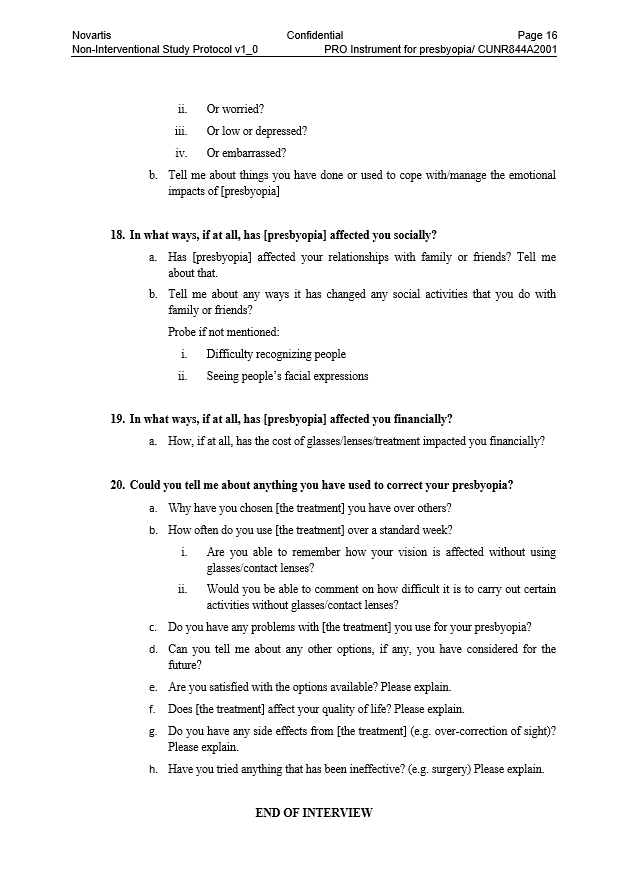

Supplement: Supplementary file 1 — Additional file 1. Interview guide for individuals with presbyopia. [file 41687_2021_383_MOESM1_ESM.docx]
