# Supplementary material for: Understanding the visual function symptoms and associated functional impacts of phakic presbyopia
Source: J Patient Rep Outcomes. 2021 Nov 3;5:114. doi: 10.1186/s41687-021-00383-1 (PMC8566618; doi:10.1186/s41687-021-00383-1)
Supplement: Supplementary file 2 — Additional file 2. Conceptual saturation. [file 41687_2021_383_MOESM2_ESM.docx]

## Appendix B

| Table 1. Conceptual saturation analysis for near vision symptoms | | | | | | | | | | | | | | | | | | | | | | | | | | | | | | | | | | | | | | | | | | | | | | | | | | |
| --- | --- | --- | --- | --- | --- | --- | --- | --- | --- | --- | --- | --- | --- | --- | --- | --- | --- | --- | --- | --- | --- | --- | --- | --- | --- | --- | --- | --- | --- | --- | --- | --- | --- | --- | --- | --- | --- | --- | --- | --- | --- | --- | --- | --- | --- | --- | --- | --- | --- | --- |
| Concept | Set 1 (n=10) | | | | | | | | | | Set 2 (n=10) | | | | | | | | | | Set 3 (n=10) | | | | | | | | | | Set 4 (n=10) | | | | | | | | | | Set 5 (n=10) | | | | | | | | | |
|  | F52-MOD-US1 | M59-MILD-US2 | F40-MILD-US3 | F52-MOD-US4 | M55-MILD-US5 | F45-MILD-US6 | M44-MOD-US7 | M41-MILD-US8 | F61-MOD-US9 | F43-MILD-US10 | F47-MILD-US11 | M54-MILD-US12 | F52-MOD-US13 | F53-MOD-US14 | M46-MILD-US15 | F58-MOD-R1-FR2 | F48-MILD-R1-FR3 | M51-MOD-R1-FR1 | M63-MOD-R1-DE1 | M61-MOD-R1-DE2 | M53-MILD-R1-DE3 | F41-MILD-R1-FR4 | F65-MOD-R1-FR5 | F50-MOD-R1-DE4 | F55-MOD-R1-DE5 | F42-MILD-R1-US1 | F54-MILD-R1-US5 | M55-MILD-R1-US2 | M60-MILD-R1-US7 | M65-MOD-R1-US6 | M45-MOD-R1-US4 | F55-MILD-R1-US3 | M65-MOD-R2-US7 | F42-MOD-R2-US3 | F60-MOD-R2-US4 | F57-MOD-R2-US5 | F41-MOD-R2-US2 | M65-MOD-R2-US6 | M50-MOD-R2-US8 | F48-MOD-R2-US1 | F58-MOD-R2-FR2 | M47-MILD-R2-DE2 | F62-MOD-R2-FR4 | F44-MILD-R2-DE3 | F40-MILD-R2-DE1 | F61-MOD-R2-FR5 | F49-MILD-R2-DE5 | F49-MILD-R2-DE4 | F59-MOD-R2-FR1 | M56-MILD-R2-FR6 |
| Impaired near vision acuity | **S** | **S** | **S** | **S** | **S** | **S** | **S** | **P** | **S** | **S** | **S** | **S** | **S** | **S** | **S** | **S** | **S** | **S** | **S** | **S** | **S** | **S** | **S** | **P** | **S** | **S** | **S** | **S** | **S** | **S** | **S** | **S** | **S** | **S** | **S** | **S** | **P** | **S** | **S** | **S** | **S** | **S** | **S** | **S** | **S** | **S** | **P** | **P** | **S** | **S** |
| Difficulty with near vision in dim light | **S** | **S** | **S** | **S** | **S** | **P** | **P** | **-** | **S** | **S** | **S** | **S** | **-** | **-** | **S** |  |  | **P** | **S** | **P** | **P** | **S** | **P** | **P** | **P** |  | **S** | **P** |  |  | **P** | **S** | **P** | **P** | **S** | **S** | **P** | **S** | **S** | **S** | **S** | **S** | **S** | **P** | **P** | **S** | **P** | **S** | **S** | **P** |
| Focusing at close distances |  | **S** |  |  | **S** |  |  |  | **P** |  |  | **S** | **P** |  |  | **P** | **P** | **P** | **-** | **P** | **P** | **S** | **S** |  | **P** | **S** | **-** | **-** | **P** | **P** | **P** | **-** | **P** | **S** | **P** | **P** | **S** | **P** | **P** | **S** | **P** | **S** |  |  | **P** | **P** | **P** | **-** | **-** | **P** |
| Difficulty seeing things in near vision when glare is present | **S** |  | **S** | **S** |  |  | **S** | **S** |  |  |  |  |  | **S** |  | **-** | **P** |  | **P** | **P** | **P** |  |  |  | **P** |  | **-** |  |  | **P** | **P** | **P** | **S** | **S** | **P** | **S** | **-** | **P** | **P** | **S** | **P** | **P** | **P** | **P** | **P** | **-** | **P** | **P** | **P** | **P** |
| Difficulty seeing contrasts in near vision | **S** | **-** | **P** | **P** | **P** | **P** | **P** |  | **P** | **P** | **-** | **P** | **-** | **S** | **-** | **P** | **-** |  | **-** | **-** | **P** | **-** | **P** | **-** | **P** | **P** | **-** | **P** | **-** | **P** | **P** | **-** | **P** | **-** | **P** | **P** | **-** | **P** | **P** | **P** | **S** | **-** | **-** | **P** | **P** | **-** | **-** | **-** |  | **P** |
| Longer time to adjust when distance of vision changes | **S** |  |  |  |  | **S** |  | **S** |  |  |  |  | **S** |  | **S** | **-** | **-** | **S** |  |  | **S** | **S** |  |  |  | **-** | **-** | **-** | **-** | **P** |  |  | **P** | **P** | **S** | **P** | **-** | **P** | **P** | **P** | **-** | **P** | **-** | **-** | **P** | **-** | **P** | **-** | **-** | **P** |
| Difficulty with near vision in bright light |  | **-** | **S** |  | **-** | **-** | **P** |  | **S** | **-** | **-** | **-** | **-** |  | **-** |  |  |  | **P** |  | **P** |  | **S** | **P** | **P** |  |  | **-** |  |  | **S** | **-** | **S** | **-** | **P** | **S** |  | **P** | **P** | **S** | **S** | **P** |  | **-** | **P** | **-** | **-** | **P** | **-** | **P** |
| Double vision |  |  |  |  |  |  |  |  |  |  | **S** |  |  |  |  |  |  |  |  |  |  |  |  |  |  |  |  | **S** |  |  |  | **S** |  |  |  | **S** |  |  |  | **S** |  |  |  |  |  |  |  |  |  |  |
| Difficulty with depth perception |  |  |  |  | **S** |  |  |  |  |  |  |  |  |  |  |  |  |  |  |  |  |  |  |  |  |  |  |  |  |  |  |  |  |  |  |  |  | **S** |  |  |  |  |  |  |  |  |  |  |  |  |
| Eye strain |  | **-** | **P** | **P** | **P** | **S** | **P** | **P** | **S** | **S** | **S** | **S** | **-** |  | **P** | **P** |  |  | **S** |  | **P** | **P** | **P** | **P** | **P** | **S** | **S** | **S** | **P** | **S** | **S** | **-** | **P** | **S** | **P** |  | **P** |  | **P** |  |  | **S** | **P** | **P** | **P** | **S** | **P** |  | **P** | **P** |
| Dry eyes | **S** |  | **S** | **S** | **P** | **S** | **-** | **P** | **P** | **P** | **-** | **-** | **-** | **S** | **-** | **S** |  | **S** | **S** | **-** | **S** |  | **P** | **-** | **P** | **P** | **S** | **S** | **-** | **P** | **S** | **P** |  | **P** | **P** | **P** | **P** | **S** |  | **-** | **P** | **P** | **P** | **P** | **P** | **P** | **P** | **P** | **-** | **P** |
| Headache | **S** | **-** | **S** | **-** | **-** | **S** | **S** | **-** | **-** | **P** | **-** | **-** | **S** | **S** | **-** | **P** | **S** | **-** | **S** | **-** |  | **P** | **-** | **-** | **P** | **S** | **S** | **S** | **P** | **P** | **S** | **-** | **S** | **S** | **S** | **S** | **S** | **P** | **P** | **S** | **P** | **-** | **-** | **S** | **P** | **-** | **-** | **-** | **-** | **S** |
| Eye irritation | **S** |  |  |  |  |  |  |  | **S** |  | **-** | **-** | **-** | **S** |  |  | **S** | **S** |  | **-** |  | **P** |  |  |  |  |  |  | **-** | **P** |  |  |  | **P** | **P** | **P** |  | **P** | **P** | **-** | **P** |  | **S** | **P** | **P** |  | **P** | **P** | **-** |  |
| Tired eyes | **S** | **-** | **-** | **S** | **P** | **P** | **-** | **S** | **-** | **S** | **-** | **-** | **-** |  | **-** | **-** |  |  | **P** | **-** | **S** |  | **S** |  |  | **P** | **P** | **S** | **-** | **-** | **S** | **-** | **P** |  |  |  |  |  |  |  |  |  |  |  | **P** | **S** | **P** |  |  |  |
| Fatigue/tiredness |  | **-** | **-** |  |  |  | **-** |  | **-** |  | **-** | **-** | **-** |  | **-** | **-** | **S** | **S** |  | **-** |  |  |  |  | **P** |  |  |  | **-** | **-** |  | **-** |  | **-** | **-** | **P** |  | **P** | **P** | **-** | **P** | **-** | **S** | **P** |  | **S** | **P** | **-** |  | **P** |
| Watery eyes |  |  |  |  |  |  |  |  |  |  |  |  |  |  |  |  |  |  | **S** | **S** |  |  |  |  | **S** |  |  |  |  |  |  |  | **S** | **S** |  |  |  |  |  |  |  |  |  |  |  |  | **S** |  |  |  |
| Eye pain |  |  |  |  |  |  |  |  |  |  |  |  |  |  |  |  |  |  |  |  |  |  |  |  |  |  |  | **S** |  |  |  |  | **S** | **S** |  | **S** |  |  |  |  |  |  |  |  |  |  |  |  |  |  |
| Dizziness |  |  |  |  |  |  |  |  |  |  |  |  |  |  |  |  |  |  |  |  |  | **S** |  |  |  |  |  |  |  |  | **S** |  |  |  | **S** |  |  | **S** |  |  |  |  |  |  |  |  |  |  |  |  |
| *Note, ‘tired eyes’ and ‘fatigue/tiredness’ were both probed as ‘fatigue’ during the interviews. During data analyses this information was separated into the two distinct symptoms. Patients who reported that this symptom was not relevant during interviews were assumed to not experience neither ‘tired eyes’ nor ‘fatigue/tiredness’. | | | | | | | | | | | | | | | | | | | | | | | | | | | | | | | | | | | | | | | | | | | | | | | | | | |

| Table 2. Conceptual saturation analysis of impact concepts | | | | | | | | | | | | | | | | | | | | | | | | | | | | | | | | | | | | | | | | | | | | | | | | | | |
| --- | --- | --- | --- | --- | --- | --- | --- | --- | --- | --- | --- | --- | --- | --- | --- | --- | --- | --- | --- | --- | --- | --- | --- | --- | --- | --- | --- | --- | --- | --- | --- | --- | --- | --- | --- | --- | --- | --- | --- | --- | --- | --- | --- | --- | --- | --- | --- | --- | --- | --- |
| Concept | Set 1 (n=10) | | | | | | | | | | Set 2 (n=10) | | | | | | | | | | Set 3 (n=10) | | | | | | | | | | Set 4 (n=10) | | | | | | | | | | Set 5 (n=10) | | | | | | | | | |
|  | F52-MOD-US1 | M59-MILD-US2 | F40-MILD-US3 | F52-MOD-US4 | M55-MILD-US5 | F45-MILD-US6 | M44-MOD-US7 | M41-MILD-US8 | F61-MOD-US9 | F43-MILD-US10 | F47-MILD-US11 | M54-MILD-US12 | F52-MOD-US13 | F53-MOD-US14 | M46-MILD-US15 | F58-MOD-R1-FR2 | F48-MILD-R1-FR3 | M51-MOD-R1-FR1 | M63-MOD-R1-DE1 | M61-MOD-R1-DE2 | M53-MILD-R1-DE3 | F41-MILD-R1-FR4 | F65-MOD-R1-FR5 | F50-MOD-R1-DE4 | F55-MOD-R1-DE5 | F42-MILD-R1-US1 | F54-MILD-R1-US5 | M55-MILD-RS-US2 | M60-MILD-R1-US7 | M65-MOD-R1-US6 | M45-MOD-R1-US4 | F55-MILD-R1-US3 | M65-MOD-R2-US7 | F42-MOD-R2-US3 | F60-MOD-R2-US4 | F57-MOD-R2-US5 | F41-MOD-R2-US2 | M65-MOD-R2-US6 | M50-MOD-R2-US8 | F48-MOD-R2-US1 | F58-MOD-R2-FR2 | M47-MILD-R2-DE2 | F62-MOD-R2-FR4 | F44-MILD-R2-DE3 | F41-MILD-R2-DE1 | F61-MOD-R2-FR5 | F49-MILD-R2-DE5 | F49-MILD-R2-DE4 | F59-MOD-R2-FR1 | M56-MILD-R2-FR6 |
| **Proximal impacts** |  |  |  |  |  |  |  |  |  |  |  |  |  |  |  |  |  |  |  |  |  |  |  |  |  |  |  |  |  |  |  |  |  |  |  |  |  |  |  |  |  |  |  |  |  |  |  |  |  |  |
| Reading | **S** | **S** | **S** | **S** | **S** | **S** | **P** | **-** | **S** | **S** | **S** | **S** | **S** | **S** | **S** | **S** | **S** | **S** | **S** | **S** | **S** | **S** | **P** | **S** | **P** | **S** | **S** | **S** | **S** | **S** | **S** | **S** | **S** | **S** | **S** | **S** | **S** | **S** | **S** | **S** | **S** | **S** | **S** | **P** | **S** | **S** | **S** | **S** | **S** | **S** |
| Seeing objects | **S** | **S** | **P** | **S** | **S** | **P** | **P** | **-** | **S** | **S** | **S** | **P** | **S** | **P** | **S** | **S** | **S** | **P** | **S** | **S** | **P** | **S** | **P** | **S** | **S** | **S** | **S** | **P** | **P** | **S** | **P** | **-** | **P** | **S** | **S** | **S** | **S** | **S** | **S** | **S** | **S** | **P** | **S** | **P** | **S** | **S** | **S** | **S** | **S** | **S** |
| **Activities of daily life** |  |  |  |  |  |  |  |  |  |  |  |  |  |  |  |  |  |  |  |  |  |  |  |  |  |  |  |  |  |  |  |  |  |  |  |  |  |  |  |  |  |  |  |  |  |  |  |  |  |  |
| Using a phone | **S** | **P** | **S** | **S** | **S** | **S** | **S** | **-** | **P** | **S** | **P** | **S** | **S** | **S** | **P** | **P** | **S** | **P** | **P** | **P** | **S** | **S** | **S** | **S** | **P** | **P** | **S** | **S** | **S** | **P** | **P** | **-** | **P** | **P** | **S** | **P** | **S** | **P** | **-** | **S** | **P** | **P** | **S** | **P** | **P** | **S** | **P** | **P** | **-** | **S** |
| Using a computer | **S** | **-** | **S** | **S** | **P** | **S** | **S** |  | **S** | **S** | **S** | **S** | **S** | **S** | **S** | **S** | **S** | **S** | **S** | **P** | **S** | **S** | **S** | **P** | **P** | **P** | **P** | **S** | **P** | **S** | **S** | **S** | **-** | **S** | **S** | **P** | **S** | **S** | **S** | **-** | **S** | **-** | **S** | **P** | **S** | **P** | **-** | **S** | **-** | **S** |
| Using a tablet device |  |  | **P** | **S** | **P** |  |  |  | **-** | **S** |  |  |  | **-** | **-** | **P** | **-** |  |  |  | **P** | **S** |  | **-** |  | **P** | **P** | **-** |  | **P** |  |  | **-** | **P** | **P** | **S** | **P** |  | **-** | **P** | **P** |  | **-** |  | **P** | **P** |  | **-** | **-** | **P** |
| Driving | **S** | **-** | **S** | **S** | **S** | **-** | **S** |  | **P** | **S** | **S** | **-** | **-** | **-** | **-** | **S** |  |  | **S** | **S** | **S** | **S** | **P** | **S** | **S** | **-** | **-** |  | **-** | **P** | **S** | **S** | **-** | **S** | **-** | **S** | **S** | **P** | **S** | **S** | **S** | **S** | **S** | **S** | **S** | **P** | **P** | **S** | **-** | **P** |
| Precision work | **S** | **S** |  |  | **S** |  | **S** |  | **S** |  |  |  |  |  |  | **S** |  |  | **P** | **S** |  |  | **S** | **S** | **P** |  | **S** |  |  | **P** |  |  |  | **S** | **S** |  | **P** |  | **S** |  | **S** | **-** | **S** |  | **S** | **S** | **S** | **S** | **S** | **S** |
| Watching TV |  |  | **P** |  |  | **S** | **P** |  | **P** | **P** | **S** |  | **S** | **S** |  | **P** |  |  | **S** | **S** | **S** |  | **S** | **P** | **P** |  |  | **P** |  |  | **S** |  |  | **S** | **S** |  | **S** | **S** |  | **S** | **P** |  |  |  | **S** |  |  |  |  |  |
| Cooking food | **S** | **P** | **-** | **-** | **P** | **P** | **-** |  | **P** | **-** | **-** | **-** | **S** | **S** | **-** | **S** |  |  | **S** | **-** |  | **S** |  | **S** | **P** | **P** | **S** | **-** | **-** | **P** |  | **-** | **-** | **-** | **S** | **P** | **S** | **-** | **-** | **P** | **S** | **-** | **-** | **-** |  | **-** | **-** |  |  |  |
| Seeing a wrist watch |  |  |  | **P** | **S** |  | **P** |  | **P** | **P** |  |  | **P** | **P** |  |  |  |  | **P** | **P** | **P** |  |  | **P** | **P** |  |  |  | **P** |  |  | **P** |  | **P** |  | **P** | **P** | **-** |  | **P** | **P** | **-** | **-** |  | **-** | **P** | **-** | **-** | **-** | **-** |
| Shopping | **S** | **S** |  | **S** |  |  |  |  |  |  |  |  |  |  |  | **S** |  |  | **S** |  |  | **S** | **S** | **S** | **S** |  |  |  | **S** |  |  |  |  |  | **S** |  |  | **S** | **S** |  | **S** | **S** | **S** |  |  |  |  |  |  |  |
| Sports/  exercise |  |  | **S** | **S** | **S** |  | **S** |  |  |  |  |  | **S** |  | **S** | **S** | **S** |  | **S** | **S** |  | **S** |  |  |  | **S** |  |  |  |  |  |  |  |  |  | **-** | **-** | **S** |  | **-** | **S** | **-** | **-** |  |  | **-** | **-** | **-** |  | **-** |
| Hobbies | **S** |  |  |  | **P** | **P** |  |  | **P** |  |  |  |  |  |  | **S** |  |  |  |  | **S** |  |  |  | **P** |  |  |  |  | **S** |  |  |  | **P** | **S** | **-** | **-** | **P** |  | **-** | **P** | **-** | **-** | **-** | **P** |  | **-** | **-** | **-** | **S** |
| Writing |  |  |  |  |  |  | **-** |  |  | **P** | **P** |  | **S** | **S** |  |  | **-** | **P** |  |  |  |  |  |  | **P** | **S** |  | **P** |  | **S** |  |  |  | **-** |  | **S** |  |  |  |  |  | **-** |  |  |  |  | **S** | **-** |  | **S** |
| Self-care |  |  |  | **P** |  |  |  |  | **P** |  | **P** |  | **P** |  |  |  |  |  |  |  |  |  |  |  |  |  | **S** |  |  |  |  |  |  | **-** | **P** | **P** | **-** |  |  | **-** |  | **-** |  | **-** | **P** |  | **S** |  | **P** |  |
| Seeing photographs |  |  | **P** |  |  |  |  |  |  |  | **S** |  | **P** | **S** |  |  |  |  |  |  |  |  |  |  |  |  |  |  |  |  |  |  |  |  |  |  |  |  |  |  |  |  |  |  |  |  |  |  |  |  |
| Walking |  |  |  |  | **S** |  |  |  |  |  |  |  |  |  |  |  |  |  |  |  |  | **S** |  | **P** |  |  |  |  |  | **S** |  |  | **S** | **S** | **S** |  |  | **S** | **S** |  |  |  |  |  |  |  |  |  |  |  |
| Tasks around home |  |  |  |  | **S** |  |  |  |  |  |  |  | **S** |  |  |  |  |  |  |  |  |  |  |  |  |  |  |  |  |  |  |  | **S** |  |  | **S** |  |  | **S** |  | **P** | **-** |  |  |  |  |  |  |  |  |
| Preparation | **S** | **S** |  | **S** |  |  |  |  |  |  |  |  |  |  |  |  |  |  |  |  |  |  |  |  |  |  |  |  | **S** |  |  |  |  |  |  |  |  |  |  |  |  |  |  |  |  |  |  |  |  |  |
| Seeing photographs |  |  | **P** |  |  |  |  |  |  |  | **S** |  | **P** | **S** |  |  |  |  |  |  |  |  |  |  |  |  |  |  |  |  |  |  |  |  |  |  |  |  |  |  |  |  |  |  |  |  |  |  |  |  |
| Seeing a clock |  |  |  |  |  |  |  |  |  | **S** |  |  | **S** |  |  |  |  |  |  |  |  |  |  |  |  |  |  |  |  |  |  |  |  |  |  |  |  |  |  |  |  |  |  |  |  |  |  |  |  |  |
| Gardening |  |  |  |  |  |  |  |  |  |  |  |  | **S** |  |  |  |  |  |  |  |  |  |  |  |  |  |  |  |  |  |  |  |  |  |  |  |  |  |  |  | **S** | **-** |  |  |  |  |  |  |  |  |
| **Distal impacts** |  |  |  |  |  |  |  |  |  |  |  |  |  |  |  |  |  |  |  |  |  |  |  |  |  |  |  |  |  |  |  |  |  |  |  |  |  |  |  |  |  |  |  |  |  |  |  |  |  |  |
| **Emotional** | **S** | **S** | **-** | **S** | **S** | **S** | **S** | **S** | **S** | **-** | **S** | **-** | **S** | **S** | **S** | **S** | **S** | **S** | **P** |  | **S** | **P** | **S** | **S** | **S** | **S** | **S** | **S** | **S** | **S** | **S** | **S** | **S** | **P** | **-** | **S** | **-** | **S** | **S** | **S** | **S** | **S** | **S** | **-** | **S** | **-** | **-** | **-** |  | **S** |
| **Work** | **S** | **S** | **S** | **S** | **S** | **P** | **S** | **-** | **S** | **P** |  | **S** | **S** | **S** | **S** | **S** | **S** | **S** |  | **-** | **-** | **S** |  | **P** | **S** | **S** | **-** | **-** | **-** | **P** |  | **S** | **-** | **S** | **S** | **S** | **S** | **P** | **S** | **-** | **S** | **P** | **P** | **-** | **S** | **S** | **S** | **P** | **-** | **S** |
| **Social** | **P** | **P** | **-** | **S** | **P** | **-** | **-** | **-** | **P** | **-** | **-** | **-** |  | **S** | **-** | **-** | **-** | **-** | **S** | **-** | **-** | **P** | **S** | **P** | **S** | **-** | **-** | **-** | **-** | **S** | **S** |  | **-** | **-** | **-** | **S** |  |  | **S** | **S** | **S** | **-** | **-** | **-** | **-** | **S** | **-** | **-** | **-** | **P** |
| **Financial** | **S** | **-** | **-** | **-** | **-** | **P** | **-** | **-** | **P** | **-** | **P** | **-** | **-** | **-** | **P** | **P** | **P** | **-** | **P** |  | **-** | **P** | **P** | **-** |  | **-** | **S** | **-** | **-** | **-** | **P** | **-** | **P** | **-** | **-** |  | **-** |  |  | **-** | **S** | **-** | **-** | **-** | **P** | **-** | **P** | **-** | **P** | **-** |
| *’S’=reported spontaneously, ‘P’=reported when probed by the interviewer, ‘-‘ = reported not relevant when probed by the interviewer. | | | | | | | | | | | | | | | | | | | | | | | | | | | | | | | | | | | | | | | | | | | | | | | | | | |
