# Supplementary material for: Understanding the visual function symptoms and associated functional impacts of phakic presbyopia
Source: J Patient Rep Outcomes. 2021 Nov 3;5:114. doi: 10.1186/s41687-021-00383-1 (PMC8566618; doi:10.1186/s41687-021-00383-1)
Supplement: Supplementary file 3 — Additional file 3. Additional primary near vision functioning symptom findings. [file 41687_2021_383_MOESM3_ESM.docx]

**Appendix C**

Further detail regarding the primary near vision functioning symptoms (Table 1) and proximal impacts (Table 2) reported by five or more individuals with presbyopia are provided below.

**Table 1.** **Primary near vision functioning symptoms reported by individuals with presbyopia**

| Symptom (n, %) S=spontaneous, P=probed | Key findings | Example supporting quotes |
| --- | --- | --- |
| **Impaired near vision acuity**  (n=50/50, 100%)  S=45 P=5 | Participants commonly used a variation of the term ‘blurry’ (n=23/50, 46%) or referred to ‘difficulty seeing’ (n=17/50, 34%) when describing their impaired near vision acuity.  Strategies to cope with impaired near vision acuity were reported by more than half of participants (n=27/50, 54%), with the majority of those reporting the use of glasses (18/50, 36%).  Scenarios where the symptom is most prominent was reported by one-third of participants (n=16/50, 32%): dim-lighting conditions (n=5/16, 31%), when reading for long periods of time (n=4/16, 25%), when using technology (n=2/16, 13%), and first thing in the morning (n=2/16, 13%). | *“I noticed, um, having to pull like nutrition labels on products in the grocery store out farther to be able to read them, um, due to the finer print.”* (F52-MOD-US1)  *“I just wear the glasses and just close my eyes and …get through it, ten more seconds.”* (M44-MOD-US7)  *“Probably just because I’ve been reading a lot or looking at a computer, on my phone.”* (F47-MILD-US11) |
| **Difficulty with near vision in dim light**  (n=42/50, 84%)  S=25  P=17 | Participants described difficulty reading in low light (n=13/42, 31%), an inability to see close-up in dark situations (n=12/42, 29%), and an inability to drive at night (n=12/42, 29%).  Participants used descriptors such as ‘blurred’ or ‘blurriness’ (n=4/42, 9%), ‘misty’ (n=1/42, 2%), ‘hazy’ (n=1/42, 2%), ‘gloomy’ (n=1/42, 2%), ‘fogged’ (n=1/42, 2%), and ‘blind’ (n=1/42, 2%) to describe their difficulty with near vision in dim light.  Strategies to cope with difficulty with near vision in dim light were reported by one-third of participants (n=14/42, 33%), with most reporting that bright lighting is needed to allow them to see in dim light (n=10/14, 71%).  Nearly one-third of participants described scenarios where the symptom is most prominent (n=12/42, 29%): at night (n=9/12, 75%) or dusk (n=4/12, 33), and when using a computer monitor (n=2/12, 17%). | *“Um, it makes, you know, dining out in dimly lit restaurants is difficult, uh, whereas I may be able to read that print, um, if it was brightly lit. I can’t when it’s dimly lit.”* (F52-MOD-US1).  *“Like my eyes, uh – I guess like they get locked in and I end up like staring sometimes at certain things just because like my eyes have like, uh, fogged and almost like I guess locked, locked for a second.”* (M44-MOD-US7).  *“Sometimes I take a flashlight and want to illuminate everything a bit more. That also helps.”* (M53-MILD-R1-DE3)  *“It's terrible at night. I try not to do any night driving 'cause I, I literally can't see.”* (F48-MOD-R2-US1) |
| **Difficulty seeing in near vision when glare is present** (n=30/50, 60%)  S=10  P=20 | Participants reported that reflection from ‘bright lights’ (n=6/30, 20%) and driving at night (n=9/30, 30%) and in certain weather conditions, such as sunlight (n=4/30, 13%) or snow (n=1/30, 3%) is bothersome.  Participants described glare as ‘blinding’ (n=4/30, 13%), ‘distracting/bothersome/ disturbing’ (n=3/30, 10%), ‘annoying’ (n=2/30, 7%), uncomfortable’ (n=2/30, 7%), ‘blurry’ (n=1/30, 3%), and ‘dazzling’ (n=1/30, 3%).  Coping strategies were described by one-third of participants (n=10/30, 33%), with most reporting the use of sunglasses to reduce the impact of glare (n=6/10, 60%). | *“Um, I think I just see a lot of halos, you know, with the oncoming traffic.”* (F52-MOD-US4)  *“I often feel blinded. Usually I also have to put on sunglasses, because I don't like brightness at all.”* (M53-MILD-R1-DE3) |
| **Difficulty focusing at close distances** (n=30/50, 60%)  S=9  P=21 | Participants described having to change position or move objects closer to focus on the detail (n=5/30, 17%) and described being unable to ‘focus on anything close’ (n=4/30, 13%).  Participants reported the symptom caused ‘straining’ (n=3/30, 10%), ‘headaches’ (n=2/30, 7%), ‘achy eyes’ (n=1/30, 3%), and ‘tired eyes’ (n=1/30, 3%).  Coping strategies were reported by several participants (n=13/30, 43%), with most reporting the use of glasses (n=9/13, 69%).  Six participants described scenarios where the symptom is most prominent (n=6/30, 20%), with most reporting that focusing at close distances is particularly challenging when reading for long periods of time (n=4/6, 67%). | *“I’ll try to focus on something close and concentrate on it to see if it’ll change, but it doesn’t. It, it’s like no, it’s not going to get any better, so put the glasses on.”* (M59-MILD-US2)  *"You know and just the other day I spent I think three hours of the day at Walmart buying groceries...You know, because I'm focusing. I have to focus and take them off and put them back on."* (M50-MOD-R2-US8) *“If I’m focused on something, I’m reading something for a long time, yes.”* (M59-MILD-US2) |
| **Low contrast sensitivity in near vision** (n=28/50, 56%)  S=3  P=25 | Participants reported difficulty seeing text on different colored backgrounds (n=9/28, 32%) and reported text is easier to read on a background with greater contrast to the text (n=5/28, 18%).  One-third of participants (n=9/28, 32%) reported scenarios where they experienced difficulties seeing low contrasts: seeing low color contrasts on receipts/bills (n=3/9, 33%), seeing low contrasts on screens (n=2/9, 22%), and seeing low contrasts at night (n=1/9, 11%).  Four participants reported adjusting the settings on digital devices to cope with difficulty seeing low contrasts in near vision (n=4/28, 14%). | *"It’s true that when letters are black on a dark background, it’s almost indecipherable."* (F58-MOD-R2-FR2)  *“Sometimes you get bills at the gas station, you can hardly read because the roll was old.”* (M53-MILD-R1-DE3)  *“I know that on, um, my Kindle, when I’m reading a book, um, no because I read at night on a black background with white print. So I think that’s okay.”* (F45-MILD-US6) |
| **Longer time to adjust when distance changes** (n=20/50, 40%)  S=9  P=11 | Seven participants discussed it takes longer to adjust their vision when distance changes (n=7/20, 35%).  Four participants reported the severity of their ability to focus at close distances (n=4/20, 20%), describing it as ‘mild-moderate’ (n=1/4, 20%), ‘minor or mild’ (n=1/4, 25%), ‘not severe’ (n=1/4, 25%), and ‘very severe’ (n=1/4, 25%).  Five participants reported coping strategies for dealing with changes in distance (n=5/20, 25%), with three reporting taking their glasses on and off (n=3/5, 60%) and two reporting squinting (n=2/5, 40%). | *“Any time to change a, a distance from reading. So like where you would normally read a book and then you look up, even the distance vision, it takes a minute to adjust to that, whereas that didn’t happen before presbyopia.”* (F52-MOD-US1)  *“Like sometimes I feel myself, you know, squinting, trying to read it without having to put my ears on.”* (F52-MOD-US1)  *“Yes, I notice that there is some kind of switch, only a very short moment, when you are not able to see anything.”* (F40-MILD-R2-DE1) |
| **Difficulty with near vision in bright light** (n=20/50, 40%)  S=8  P=12 | A few participants referred specifically to ‘difficulty seeing’ in bright light (n=3/20, 15%) and described that bright light ‘strains’ or ‘hurts’ their eyes (n=3/20, 15%)  Coping strategies were reported by one-third of participants (n=6/20, 30%), with two participants reporting use of sunglasses (n=2/6, 33%) and two reporting squinting (n=2/5, 33%) to help see in bright light.  Seven participants described scenarios where the symptom is most prominent (n=7/20, 35%): sunlight/natural light (n=4/7, 57%), looking at computer monitor (n=2/7, 29%), dark light for extended periods (n=1/7, 14%), or under bright fluorescent tube lighting (n=1/7, 14%). | *“…the brightness makes my eyes - it strains my eyes, So I, I bring it down, um, maybe like midway so that it can, can meet up… Because sometimes the light is just way too bright.”* (F40-MILD-US3)  *“Otherwise, in very bright light, put on sunglasses.”* (M53-MILD-R1-DE3)  *"For example, being blinded by high beam glares from oncoming traffic. Bright is simply when you are sitting under a bright fluorescent tube."* (F40-MILD-R2-DE1)  *"It’s less problematic than in darkness, yes, in darkness. But it’s still an issue in bright light."* (F58-MOD-R2-FR2) |

| Table 2. Proximal impacts reported by individuals with presbyopia | | |
| --- | --- | --- |
| Impact (n, %)* S=spontaneous, P=probed | Key findings | Supporting quotes |
| **Difficulty reading in near vision**  (n=49/50, 98%)  S=45  P=4 | All 49 participants reported difficulty reading printed text (n=49/50, 98%), with 19 reporting they had difficulty reading printed text specifically when it is small in size (n=19/49, 39%).  A large proportion of participants reported difficulty reading handwriting (n=34/49, 69%)  Seven participants reported that reading handwritten text differs in terms of difficulty from printed text (n=7/34, 21%), with three reporting it is more difficult to read printed text (n=3/7, 43%)  One-third of participants reported that dim lighting makes it more difficult to read (n=16/49, 33%).  Impacts included reading: menus (n=39/49, 80%), labels or ingredients (n=34/49, 69%), newspapers or magazines (n=25/49, 51%), books (n=23/49, 47%), receipts (n=18/49. 37%), documents (n=17/49, 35%), and mail (n=14/49, 29%).  Adjustments used when reading included: adjusting the distance from the text (n=10/49, 20%), asking somebody to read for them (n=9/49, 18%), using a magnifying glass (n=7/49, 14%), moving near a light/creating more light (n=6/49, 12%), and using a phone to enlarge text (n=5/49, 10%). | *“Um, I want to say notes. Sometimes when they leave notes and they write them with pencil, they're a lot of faint, fainted, so it's a little difficult to see."* (F42-MILD-R1-US1)  "*Um, it makes, you know, dining out in dimly lit restaurants is difficult, uh, whereas I may be able to read that print, um, if it was brightly lit. I can’t when it’s dimly lit*." (F52-MOD-US1)  *“Well I have to – if I have to read, I have to focus, bring the page closer to me, something like that”* (F47-MILD-US11)  “*Like right now I just went to pick up my medicine and I have to like, I have to wear my glasses to read the instructions."* (F53-MOD-US14) |
| **Seeing objects close up**  (n=48/50, 96%)  S=34  P=14 | Most participants reported they have difficulty seeing objects close up generally (n=20/48, 42%), followed by a wristwatch (n=14/48, 29%), cosmetic tasks (n=5/48, 10%), seeing objects in a store (n=4/48, 8%), and seeing objects when doing arts and crafts (n=3/48, 6%).  Nine participants discussed methods they use to see close up (n=9/48, 44%), namely a magnifying glass (n=4/9, 44%) or squinting (n=3/9, 33%). | *"So a lot of the smaller things I have to use magnifiers for, magnifying glass actually. I look like Sherlock Holmes."* (F52-MOD-US4)  “*Without a magnifying glass, yes. Like, um, tweezing my eyebrows… Maybe polishing my nails… Putting makeup on, I need a magnifying glass."* (F47-MILD-US11) |
| **Use of digital devices**  (n=48/50, 96%)  S=40  P=8 | Participants reported difficulty using a phone (n=46/49, 94%), a computer (n=43/49, 88%), watching tv (n=24/49, 50%), using a phone keypad (n=21/49, 43%), and using a tablet (n=19/49, 39%).  Less frequently mentioned digital impacts included: using a Kindle (n=5/49, 10%), computer keyboard (n=5/49, 10%), remote control (n=4/49, 8%), stylus pen (n=4/49, 8%), and camera (n=1/49, 2%).  Participants reported increasing the font size (n=32/49, 65%), using the zoom function (n=21/49, 43%), and increasing (n=13/49, 27%) or reducing (n=7/49, 14%) the brightness on their device, adjusting their distance (n=12/49, 24%), and turning their devices horizontally to enlarge content (n=11/49, 22%) to help them see more clearly. | *“Like I'll look at Netflix on my phone and stuff like that, and it's like I got to stop doing that because like I'll look at it and I'll fall asleep and then my eyes would be burning… It's just I guess the screen is so small and it's so close.”* (F41-MOD-R2-US2)  *"I notice myself having to enlarge the font on either the tablet or the phone."* (F43-MILD-US10) |
| **Driving**  (n=34/50, 68%)  S=27  P=7 | Half of the participants mentioned difficulty driving in the dark (n=17/34, 50%).  One-third of participants described difficulty seeing street signs when driving (n=11/34, 32%) and one-quarter reported difficulty seeing their dashboard (n=8/34, 24%)  A few participants described difficulty driving at night due to headlights (n=3/34, 9%) and driving in bright sunlight (n=2/34, 6%).  Two participants reported using sunglasses to help their vision when driving (n=2/34, 5.9%). | “*It's just dark. Like I can't see street signs. Um, and then, um, the headlights from the cars interfere with that too because everything is blurry. And then you add—you know, it's like it's dark of night and then you add this bright light to it. Oh, it's just terrible.”* (F48-MOD-R2-US1)  *“Um, missing turns, um, um, just basically even with the sun, it, it is a factor thing so with the sun, um, missing signs, and just trying to make sure I’m not really, really as far as or close to a car that I am*.” (F42-MOD-R2-US3) |
| **Precision work**  (n=25/50, 50%)  S=21  P=4 | Most participants reported an impact on their ability to sew or do needlework (n=19/25, 76%).  Other types of precision work impacted included: fixing things (n=6/25, 24%), measuring things (n=2/35, 8%), replacing batteries (n=2/25, 8%), using tools at work (n=2/25, 8%), painting/drywall/woodwork (n=1/25, 4%), and using computer-aided design (CAD) software (n=1/25, 4%). | *“Or if I wanted to do some very small dexterity work, if I wanted to get a thread through the eye of a needle, that would certainly be harder than it used to be. Such fine things.”* (M61-MOD-R1-DE2)  “*If I’m working on like a project that involves, uh, soldering or looking at circuit boards and things like that. That usually requires like I said glasses and then like an additional one of those, uh, giant, uh, mirror magnifying glasses you see in cinema.”* (M44-MOD-US7) |
| **Cooking**  (n=20/50, 40%)  S=11  P=9 | The most commonly reported impact on cooking reported by participants was being able to see a recipe (n=7/20, 35%).  Other impacts included: measuring ingredients (n=3/20, 15%), seeing what they’re cooking (n=1/20, 5%), finding hair in food (n=1/20, 5%), and seeing the buttons on the stove (n=1/20, 5%).  Adjustments made while cooking included turning on the stove light (n=1/20, 5%) and wearing a ‘cut glove’ to protect themselves (n=1/20, 5%). | *“Any, any cooking directions that may be, um, any—like I can’t read the measuring cup. You know, I can’t get it far enough away from me to read it.”* (F52-MOD-US13)  “*Sometimes I have to press the light in the, uh, in the hood. I have to press the light because I want to see what I’m cooking*.” (F53-MOD-US14) |
| **Seeing a wristwatch**  (n=20/50, 40%)  S=1  P=19 | Whilst 20 participants reported difficulty seeing a wristwatch, all but one participant only reported this concept only when probed.  One participant reported that they found a digital watch easier to read (n=1/20, 5%).  Three participants described adjustments they made to improve their ability to see a wristwatch: buying a larger watch (n=2/19, 11%), using their smartphone instead (n=2/19, 11%), and moving the watch further away (n=1/19, 5%) | *“You know, it’s blurry, but I know it, so how do you answer that if you know I can look down and go, okay. You know, I don’t know. Yes. I cannot see the date.”* (F52-MOD-US13)  “*I have to buy like a big one like that that I can see the numbers because if, if they’re kind of small I cannot see it*.” (F53-MOD-US14) |
| **Shopping**  (n=16/50, 32%)  S=16  P=0 | Participants reported difficulty with shopping due to reading labels/ingredients on a product (n=8/16, 50%), seeing products in front of them (n=4/16, 25%), reading tags and prices on products (n=3/16, 19%), seeing their shopping list (n=1/16, 6%), and seeing their credit card/card reader when paying (n=1/16, 6%)  Two participants reported that shopping takes them longer because of their impaired near vision (n=2/16, 13%)  Two participants reported adjustments made to help them shop (n=2/16, 13%), with one asking family members to read labels and the other asking someone else in the store to read the price of an item | “*Seeing things close … it depends on where I am, what the circumstances are, when I go into a shopping mall it’s terrible, I have the impression I can’t see well.”* (F65-MOD-R1-FR5)  “*you know, if I go, oh I’m going to go to the grocery store I might remember to bring a pair of glasses with me if I’m going to read a label.”* (M59-MILD-US2) |
| **Sports/exercise**  (n=14/50, 28%)  S=14  P=0 | Four participants reported difficulty swimming due to not being able to take their glasses in the pool with them (n=4/14, 29%).  Two participants reported difficulty riding a bike (n=2/14, 14%).  Other sports/exercise impacts reported by one participant each were: roller skating, badminton, triathlons, water skiing, deer hunting, basketball or seeing their child do sports.  These impacts were mostly due to an inability to adjust vision from near to far or not being able to see objects close up. | *“If I roll for too long, maybe like five, six, seven songs without coming off the floor, then I could be faced with like a little bit of eyestrain because like I feel like my eye was just trying to catch up with my surroundings basically.”* (F40-MILD-US3)  “*Cause I've always played sports too and that's kind of important to know where the basket is or where the ball is coming.”* (M65-MOD-R2-US6) |
| **Hobbies**  (n=14/50, 28%)  S=6  P=8 | Participants reported various hobbies that were impacted by presbyopia: arts and crafts (n=4/14, 29%), coin/stamp collecting (n=2/14, 14%), completing puzzles (n=2/14, 14%), going to the cinema (n=2/14, 14%), playing board or card games (n=2/14, 14%), knitting (n=1/14, 7%), reading sheet music for singing (n=1/14, 7%), cake decorating (n=1/14, 7%), painting/upholstering (n=1/14, 7%), doing crosswords (n=1/14, 7%), and boating (n=1/14, 7%). | *“Well I used to do paper crafting, um, as a hobby and I find I do less of it… would have to separate a segment of time where I could wear my glasses the whole time to do that paper crafting.”* (F52-MOD-US1)  “*Um, I, I do crosswords. Uh, that's, that's, that's cumbersome. That kind of slows me down because I'm not sure what I'm looking at or what I'm seeing. Um, that's not true either because if I put a pair of readers on I can adjust the page to see about anything, but, you know,”* (M65-MOD-R2-US6) |
| **Writing**  (n=12/50, 24%)  S=7  P=5 | Four participants reported the need for glasses to see what they are writing (n=4/12, 33%).  Two participants reported writing larger to help them see what they have written (n=2/12, 17%).  Ten participants reported that this impact is not relevant to them when probed (n=10/50, 20%). | *“Writing things down. Um, if I were writing things, uh, yes. Certainly, um, I might even write larger than I normally have.”* (M65-MOD-R1-US6  “*Even for me, to write, I have to put my glasses on.”* (M56-MILD-R2-FR6) |
| **Self-care**  (n=10/50, 20%)  S=2  P=8 | Six participants reported difficulty putting on make-up due to presbyopia (n=6/10, 60%), with one participant reporting she used a magnifying mirror to help with the process (n=1/10, 10%), and two participants reported difficulty tweezing their eyebrows (n=2/10, 20%).  Other self-care impacts were: doing hair (n=1/10, 10%), applying nail polish (n=1/10, 10%), manicure/pedicures (n=1/10, 10%), getting dressed (e.g., buttons) (n=1/10, 10%). | *“I was going to say probably—which is very hard to do, like to make a really good line, eyeliner. You know, like you want to keep them on just to make—you know, because it's fine work in there.”* (F54-MILD-R1-US5)  *“However I do button my clothes different. You know, one button here. So I be like, I couldn’t even see that button. You know how you*” (F57-MOD-R2-US5) |
| **Walking**  (n=9/50, 18%)  S=8  P=1 | Two participants reported that they bump into/trip over things because they are unable to see where they are walking (n=2/9, 22%).  One participant each (n=1/9, 11%) reported: the time it takes for vision to adjust to different distances makes it challenging to walk without contact lenses, difficulty seeing where he/she is walking in dim lighting, and feeling uncomfortable walking at night due to poor near vision. | *“Well, walking, quite simply, since looking at the ground and looking in front of me – since I have, as I said, this instability issue, the mere fact of walking … just living, quite simply.”* (F41-MILD-R1-FR4)  *“Well, I know this much, in dim lighting I don't see well, and ah because I’ll fall over something in a minute in dim light because I can’t see it, you know*.” (M65-MOD-R2-US7) |
| **Tasks around the home**  (n=6/50, 12%)  S=5  P=1 | Participants described various ways presbyopia impacts their ability to complete tasks around the home: cleaning (n=3/6, 50%), repairing things (n=1/6, 17%), using a screw driver (n=1/6, 17%), opening a safe (n=1/6, 17%), and reaching for objects (n=1/6. 17%). | *“I’m redoing my house, so I was putting up doors, um, hanging doors, so the screws—although that’s a big hinge that goes on it, I still need my glasses to be able to screw, screw stuff in. And, um, cleaning, cleaning. Um, you know, really close up. I can see dirt a lot better with the glasses, you know.”* (F52-MOD-US13) |
